# Selenium nanoparticles for targeted stroke therapy through modulation of inflammatory and metabolic signaling

**DOI:** 10.1038/s41598-019-42633-9

**Published:** 2019-04-15

**Authors:** Hamed Amani, Rouhollah Habibey, Fereshteh Shokri, Seyed Javad Hajmiresmail, Omid Akhavan, Alireza Mashaghi, Hamidreza Pazoki-Toroudi

**Affiliations:** 1 Department of medical nanotechnology, Faculty of Advanced Technologies in Medicine, Iran University of Medical Science, Tehran, Iran; 2 Department of Neuroscience and Brain Technologies-Istituto Italiano di Technologia, Via Morego, Genova, Italy; 3 International Pharmaceutical Federation, The Hague, Netherlands; 4 Department of Cardiology, Iran University of Medical Sciences, Tehran, Iran; 5 Department of Physics, Sharif University of Technology, Tehran, Iran; 6 Leiden Academic Centre for Drug Research, Faculty of Science, Leiden University, Leiden, Netherlands; 7 Harvard Medical School, Harvard University, Boston, USA; 8 Physiology Research Center and Department of Physiology, Faculty of Medicine, Iran University of Medical Sciences, Tehran, Iran

## Abstract

Ischemic cerebral stroke is a major cause of death and morbidity. Currently, no neuroprotective agents have been shown to impact the clinical outcomes in cerebral stroke cases. Here, we report therapeutic effects of Se nanoparticles on ischemic stroke in a murine model. Anti-transferrin receptor monoclonal antibody (OX26)-PEGylated Se nanoparticles (OX26-PEG-Se NPs) were designed and synthesized and their neuroprotective effects were measured using *in vitro* and *in vivo* approaches. We demonstrate that administration of the biodegradable nanoparticles leads to resolution of brain edema, protection of axons in hippocampus region, and myelination of hippocampal area after cerebral ischemic stroke. Our nanoparticle design ensures efficient targeting and minimal side effects. Hematological and biochemical analyses revealed no undesired NP-induced changes. To gain mechanistic insights into the therapeutic effects of these particles, we characterized the changes to the relevant inflammatory and metabolic signaling pathways. We assessed metabolic regulator mTOR and related signaling pathways such as hippo, Ubiquitin-proteasome system (ERK5), Tsc1/Tsc2 complex, FoxO1, wnt/β-catenine signaling pathway. Moreover, we examined the activity of jak2/stat3 signaling pathways and Adamts1, which are critically involved in inflammation. Together, our study provides a promising treatment strategy for cerebral stroke based on Se NP induced suppression of excessive inflammation and oxidative metabolism.

## Introduction

Cerebral stroke has been considered as a major health concern^1^. By 2050 a growing number of people around the world will be aged 65 years or older which would lead to an increase in age related diseases including the stroke. Intense research is currently underway for developing preventive strategies, understanding the disease dynamics and mechanism, as well as for treatment of stroke and its associated disabilities^2,3^. Developing drugs that target the central nervous system (CNS) remains a major challenge for pharmaceutical science and industry. Fast metabolization, clearance from blood circulation and poor transport across the blood-brain barrier hamper the efficacy of most central nervous system drugs^4^. A major challenge in developing anti-stroke drugs is the complexity of the signaling processes involved as well as the associated inflammatory response that further complicates the problem. In the mammalian CNSs, signaling networks including mTOR, Wnt/β-Catenin, Hippo-Yap-Mst, Jak-Stat affect neuronal growths and metabolisms^5,6^; dysregulation of these pathways during or after stroke results in neuronal damage and death^7^. Moreover, hippocampal neurogenesis, synaptic plasticity and memory consolidation are inhibited by pro-inflammatory cytokines^8^. Understanding these pathways enables rational targeting of selected pathways and leads to efficient therapeutic strategies with minimal side effects. Intense research is underway to develop nanoparticle-based strategies for targeted therapy and drug delivery^9,10^. Targeted therapy by nano drug delivery systems has garnered much attention to treat various diseases such as cancer in recent years^11–15^. Among these, Se nanoparticles emerged as promising tools for fighting major CNS disorders. Selenium (Se) is an essential trace element to human health with unique physiological and pharmacological properties which reduce the incidence of neurodegenerative diseases^16,17^. In fact, Se can participate in modulation of neurogenesis, electron transport chain dynamics, preservation of the redox balance, and regulation of the Ca^2+^ transport in the neural cells. High concentrations of the polyunsaturated fatty acids in the brain make it highly vulnerable to oxidative stress^18^. Several Se nanoparticles have been developed and their applicability for neurological diseases has been tested. For instance, Epigallocatechin-3-gallate (EGCG)-stabilized Se nanoparticles (NPs) coated with Tet-1 peptide could enhance recovery of Alzheimer’s disease through inhibition of amyloid-β aggregation, *in vitro*^19^. Se nanoparticles exhibit high antioxidant activity^20^ and selenoproteins reinforce endogenous antioxidant system. The selenoproteins are significantly expressed in the human brain and their inherent antioxidant activity contributes to the free radical defense system and brain function. The expression of selenoproteins is associated with dietary Se intake. Furthermore, Se administration contributes to mitochondrial dynamics through an autophagy dependent mechanism after focal cerebral ischemia^21^. In this work, for the first time, we synthesized and studied the capability of OX26 antibody functionalized Se NPs (with sizes ~12 nm) for *in-vivo* targeted therapy of brain ischemic stroke in a Wistar rat model. This was justified based on recent reports indicating that the transferrin receptor (TfR) is highly expressed in brain capillary endothelial cells to facilitate entry of the iron transporting protein into the brain^22^. OX26 is a monoclonal antibody against the transferrin receptors, providing targeted drug delivery to the brain through a receptor-mediated transcytosis pathway^23^. We further studied the interaction of the Se NPs with interconnected signaling pathways including Tsc1/Tsc2 complex, FoxO1, mTORC1/mTORC2, Wnt/β-Catenin, Hippo-YAP-MST, USP and autophagy after focal cerebral ischemia-reperfusion. In addition, the regulatory effects of the Se NPs on the activity of Jak2/Stat3, Mst1, mTORC1, ADAMTS1 as well as apoptosis were evaluated after the focal cerebral ischemia reperfusion. Based on these investigations, we propose a model that explains the therapeutic effects of Se NP for cerebral stroke.

## Results and Discussion

### Synthesis, characterization, stability and particle-protein interactions of OX26-PEG-Se NPs

**Figure 1:**
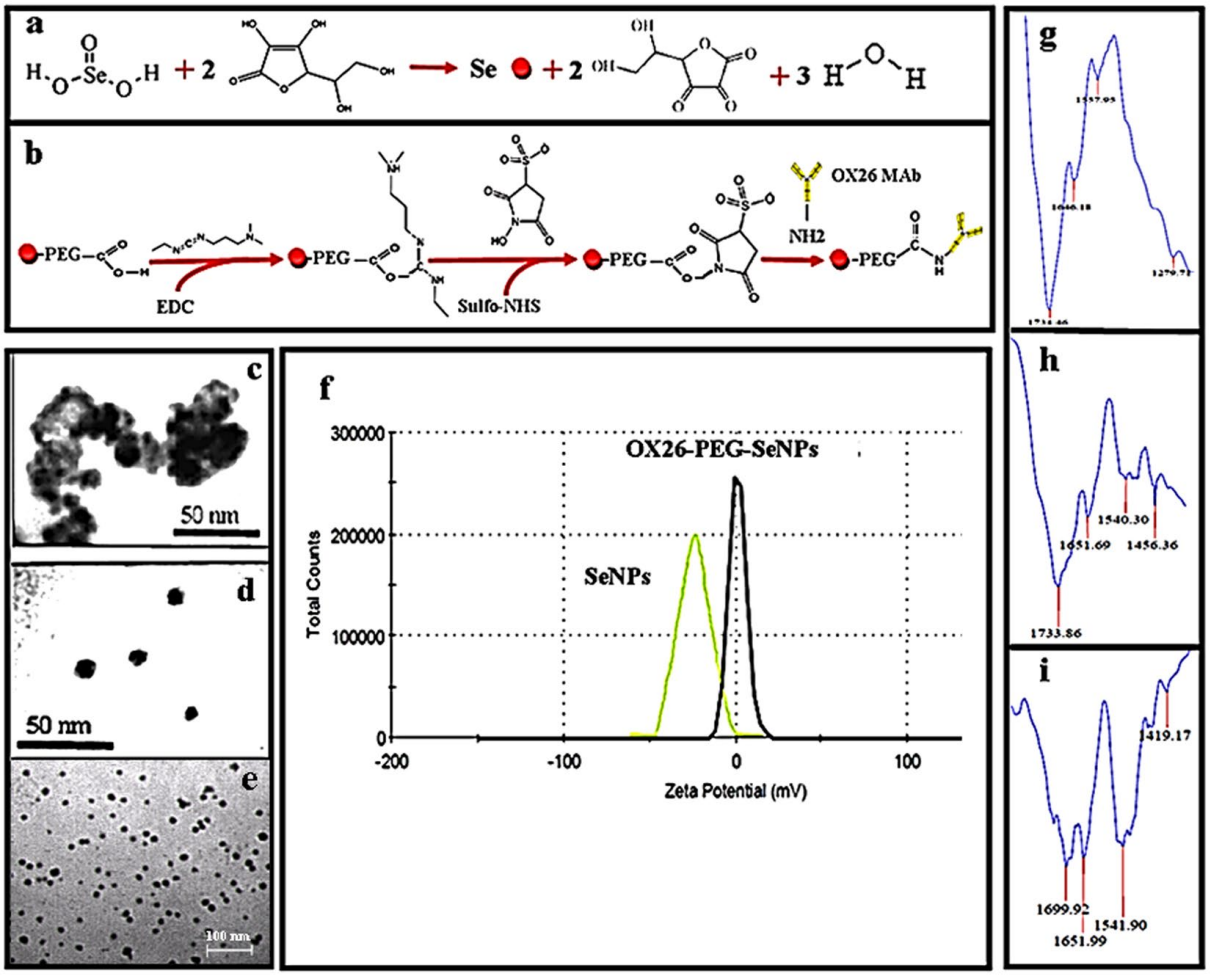
Schematic illustration of reaction steps involved in the synthesis (**a**) and functionalization (**b**) of Se NPs are given. Representative TEM images of bare (**c**) and PEGylated (**d,e**) Se NPs are shown. Panel (f) presents the zeta potential of the bare and antibody functionalized Se NPs. Panels (g–i) are FTIR spectra of bare Se NPs, PEG-Se NPs and OX26-PEG-Se NPs respectively.

Figure 1a illustrates the method used for synthesis of the Se NPs. Changes in volume of 0.1 M selenious acid was resulted in synthesis of Se NPs with different sizes (Fig. S1). Then, the Se NPs were PEGylated and functionalized by OX26 monoclonal antibody to obtain a targeting nanoparticulate system (Fig. 1b). Quantitation of the OX26 monoclonal antibody immobilized onto the surface of NPs was performed (Table S1). Various spectroscopic and microscopic methods were used to characterize the morphology, the physicochemical properties, and localization of the OX26-PEG Se NPs. Figure 1c–e presents the transmission electron microscopy (TEM) images of aggregated Se NPs and monodisperse spherical particles of OX26-PEG–Se NPs with an average diameter of ~12 nm, which is further confirmed by dynamic light scattering (DLS, polydispersity 0.05) technique. The bare Se NPs were significantly aggregated due to their high surface energy, which in turn resulted in their precipitation at the bottom of the cuvette (Figs 1c and S2). Functionalization of the bare Se NPs with PEG-OX26 significantly increased their stability. To evaluate the effects of PEG-OX26 functionalization on the surface charge of the Se NPs, bare Se NPs and OX26-PEG-Se NPs zeta potential was measured. The average surface charge of bare Se NPs was approximately −24 mV, which converted to +1.17 mV after functionalization, thus a higher cellular uptake is expected for the OX26-PEG-Se NPs compared to the bare NPs. FTIR spectroscopy was used to confirm the chemical bonds formation between OX26 antibody and PEG-Se NPs. For the OX26-PEG-Se NPs, the peak at 1699 cm^−1^ confirmed the presence of OX26 protein on surface of the Se NPs (Fig. 1g–i), consistent with the previous reports^24^.

**Figure 2:**
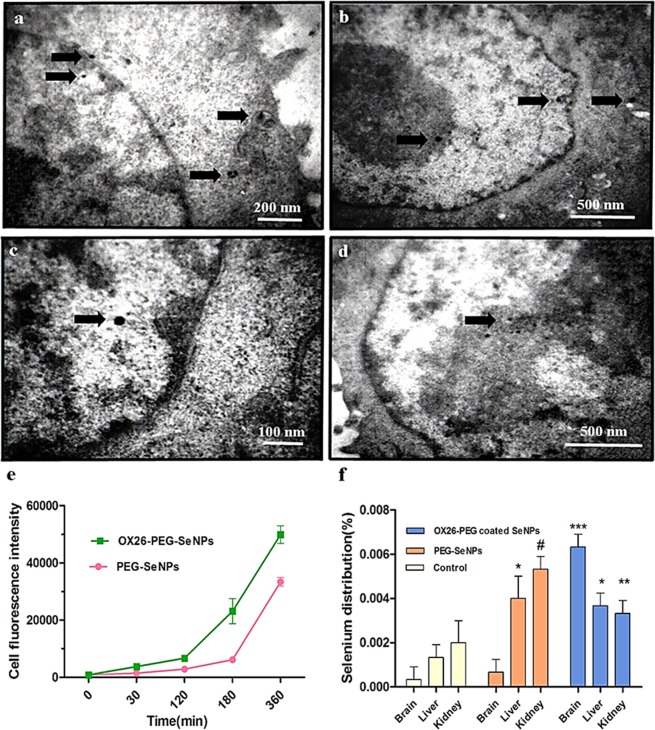
TEM images of differentiated PC12 cells exposed to 100 μg/mL of OX26-PEG-Se NPs (25 nm in size) in cMEM for 1 h (**a,b**) and 3 h (**c,d**). The arrows show the major localization of the NPs within the nucleus. (**e**) Kinetics of uptake of 50 μg/mL of FITC-OX26-PEG-SeNPs (12 nm in size) and FITC-PEG-Se NPs in cMEM by differentiated PC12 cells at different times evaluated by flowcytometry (average of three replicates ± sd). (**f**) Comparison of elemental Se distribution in different tissues 24 h after intraperitoneal administration of PEG-Se NPs and OX26-PEG-Se NPs by ICP-OES analysis. The Se content of brain significantly increased in OX26-PEG-Se NPs group compared to control and PEG-Se NPs at dose of ~1000 μg/mL. The symbols ***, ** (and also *), and # show the P-values of P < 0.001, 0.05, and 0.01 as compared to control, respectively.

Next, we tested whether PEG-functionalization can suppress corona formation on the synthesized particles. Non-specific protein adsorption on the surface of the NPs may have profound effects on stability, biocompatibility and cellular uptake^25^. Hence, we investigated the changes in size distribution, polydispersity and zeta potential of the bare Se and OX26-PEG-Se NPs in both 10% human plasma and complete medium supplemented with 10% serum (cMEM). The results of the physicochemical characterizations are summarized in Tables S2–S5. The results showed that size distribution of the bare NPs dramatically shifted to higher values due to protein adsorption on their surface, while the PEG-functionalized NPs showed significantly lower size variations, suggesting a reduced affinity of the functionalized NPs for plasma proteins. Interactions of the synthesized particles with cells were also studied and corona formation was assessed. Previous studies showed that interactions between biomolecules in the corona and corresponding receptors in the cell membrane may affect the cell uptake of NPs, and therefore the cell survival^26^. Salvati and Dawson found that NP uptake in serum free (SF) condition resulted in cell damage by a strong adhesion of Silica NPs on the cell membrane^27^. Using rat PC12 cells and the human breast cancer (MCF7) cells, we evaluated cellular uptake of FITC-OX26-PEG-Se NPs and cell survival followed by oxygen glucose deprivation (OGD) at different times (Figs S3 and S4). When the FITC-OX26-PEG-SeNPs exposed to cells in SF condition, the particle uptake, cellular damage and cell death were found to be higher in comparison to those in the cMEM condition after 120 min incubation. Severe alterations in cell shape such as cell shrinkage and disintegration of cell-to-cell contacts are clearly observed by using fluorescence microscopy after 180 min in SF condition, indicating the initiation of the apoptotic and/or necrotic cell death. In fact, we did not observe any re-growth and/or cell viability after 180 min uptake (followed by a centrifugation at 2000 rpm and re-seeding of the cell). Surprisingly, the cMEM condition decreased the cell death after the OGD. In 360 min NP uptake (which followed by a centrifugation step at 2000 rpm), the cells were seeded again. We observed a good coordination of cell growth and division, suggesting not only lower uptake in the presence of serum, but also positive effect of the serum (possibly through formation of corona layer) on the essential antioxidant activity of the NPs in culture medium having oxidative stress conditions. Overall, since the necrotic cell death and chronic inflammation are closely correlated with the inhibition of the hippocampal neurogenesis and memory dysfunction, we found suggestive evidences for a positive *in vitro* effect of protein corona on neuronal survival, under ischemic conditions. In the next step, the uptake and internalization of the NPs by the PC12 cells were studied using TEM. As shown in Fig. 2a,b, OX26-PEG-SeNPs enclosed in vesicles and transported across the cell membrane, presumably through receptor-mediated endocytosis. The NPs seemed to be engulfed in early endosome or free in the cytosol. Astonishingly, our result confirmed that the localization of the NPs was in the nucleus (Fig. 2c,d). To directly assess the targeting capabilities of the Se-based NPs for neuronal cells, we performed the following flowcytometry analysis. The PC12 cells were treated with Nerve growth factor (NGF) and 1% serum (1:100) for 5 days to differentiate into neural cells *in vitro*. When OX26-PEG-SeNPs were exposed to the cells in cMEM condition, significant uptake levels were observed for the OX26-PEG-SeNPs than the PEG-Se NPs at 37 °C at different times, indicating the targeting effect of the functionalized OX26 antibody (Fig. 2e). Then, in order to study whether the targeting capability of the OX26-PEG-SeNPs maintains *in vivo*, we evaluated the elemental Se distribution 24 h after intraperitoneal administration of the PEG-Se NPs and OX26-PEG-Se NPs by using ICP-OES analysis of brain, liver and kidney tissues (Fig. 2f). The Se content of kidney tissue was more than brain and liver in control and PEG-Se NPs groups. Strikingly, the selenium content of the brain increased compared to other tissues in OX26-PEG-Se NPs, confirming the *in vivo* targeting capabilities of the functionalized Se NPs.

### Pharmacological efficiency

**Figure 3:**
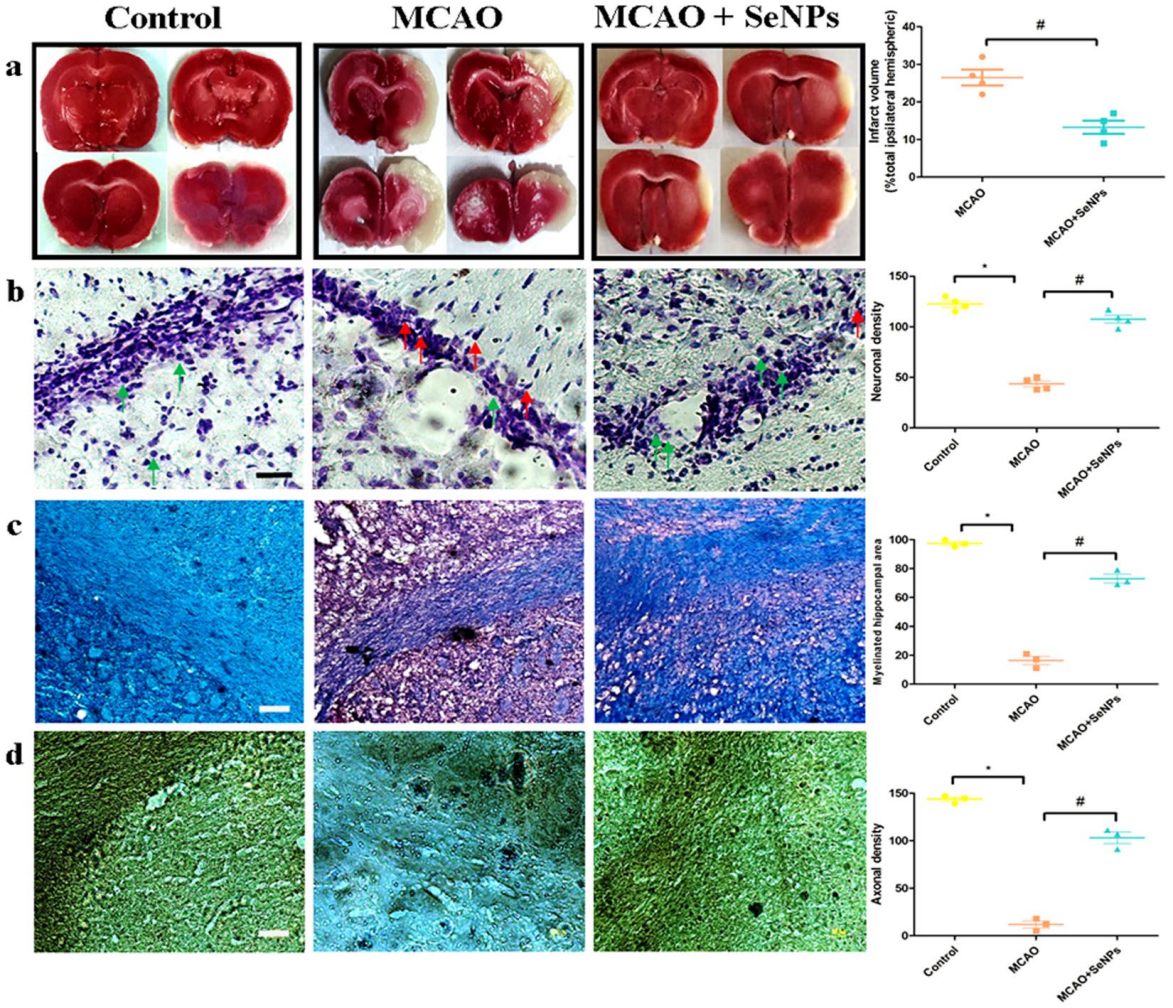
(**a**) TTC staining which presents significant decrease of infarct volume (^#^p < 0.01 compared to MCAO group) by OX26-PEG-Se NPs. The non-infarcted brain tissue appeared red and the infarcted area appeared white. (**b**) Nissl staining of hippocampal CA1 subregion. Green and red arrows indicate intact and necrotic cells, respectively. The OX26-PEG-Se NPs significantly contributed to enhancement of neuronal survival under oxidative stress (*p < 0.001 and ^#^p < 0.001 compared to control and MCAO groups, respectively). (**c**) LFB staining of hippocampal CA1 subregion. Myelinated fibers (blue), neutrophils (pink) and nerve cells (purple) are observable. The MCAO significantly decreases myelinated hippocampal area, while OX26-PEG-Se NPs administration significantly maintained myelinated hippocampal region (*p < 0.001 and ^#^p < 0.001 compared to control and MCAO groups, respectively). (**d**) Bielschowsky’s silver staining of hippocampal CA1 subregion. The OX26-PEG-Se NPs administration strongly maintained the number of axons in hippocampal CA1 subregion (*p < 0.0001 and ^#^p < 0.0001 compared to control and MCAO groups, respectively, axons: black) (All scale bars are 200 μm).

Growing evidences suggest that Se NPs can act as anti-tumor, anti-fungal and anti-bacterial agents, but it is not known whether these NPs can contribute to neuronal survival after stroke^28–31^. The major cause of neuronal cell death in ischemic brain injury is cellular swelling. Aberrant entry of Na^+^ ions increases the voltage-gated chloride channel activity, which in turn causes a sequential chain of high cytoplasmic level of sodium and chloride ions, osmotic imbalance and subsequently cytotoxic edema^32^. Here, we found that intraperitoneal administration of the OX26-PEG-SeNPs decreased the brain edema in Wistar rats subjected to 30 min of middle cerebral artery occlusion (MCAO) and 22 h of reperfusion. In our study, brain water content was significantly increased in response to focal cerebral ischemia. The OX26-PEG-SeNPs significantly decreased brain water content compared to the MCAO and vehicle groups (Fig. S5). Moreover, the infarction volume was evaluated by using 2,3,5-triphenyltetrazolium chloride (TTC) staining method (Fig. 3a). Surprisingly, the OX26-PEG-Se NPs reduced infarction volumes. Compared with the MCAO and vehicle groups the stroke size was smaller in the OX26-PEG-Se NPs group.

To evaluate if the neuroprotective effect of the OX26-PEG-Se NPs may have clinical utility, the NPs were administered 1 h after stroke and neuronal density was determined by hippocampal Nissl staining (Fig. 3b). The neuronal density significantly fell throughout the hippocampus region in the MCAO group. The percentage reduction in neuronal density was significantly lower for the OX26-PEG-Se NPs treated group, suggesting the increased neuronal cell survival by the NPs under oxidative stresses. It is known that the hippocampus is rich in myelin than other gray matter regions^33^. Hence, in order to further investigate the effects of the OX26-PEG-Se NPs on neuronal survival, we have studied myelinated hippocampal area in different groups by the Luxol fast blue (LFB) technique (Fig. 3c). A significant decrease in the LFB density on ipsilateral brain sections was observed in the MCAO group compared with baseline, indicating extensive loss of myelin in the hippocampus region. However, for the OX26-PEG-Se NPs group, the LFB indicated a significant increase of density in the ipsilateral hemisphere. Later, we investigated whether induction of the MCAO affects the number of axons in hippocampus region. To assess the number of axons, the tissue sections were stained by Bielschowsky’s silver method. A significant reduction of hippocampal axon density in the MCAO group was observed (Fig. 3d).

In contrast, the OX26-PEG-Se NPs could maintain the density of axons in hippocampus region. Parallel to the reductions in the infarction volumes, we also observed recovery of locomotor function by evaluation of neurologic deficit scores and ladder test (Fig. S6).

### Side effects and systemic toxicity

A great challenge of NP-based drugs is the probable emergence of side effects on short or long time scales^34,35^. Excessive Se is known to be associated with hepatic damage, diabetes, neurodegenerative and cardiovascular disease^36^. Therefore, to determine whether the Se-based.

**Figure 4:**
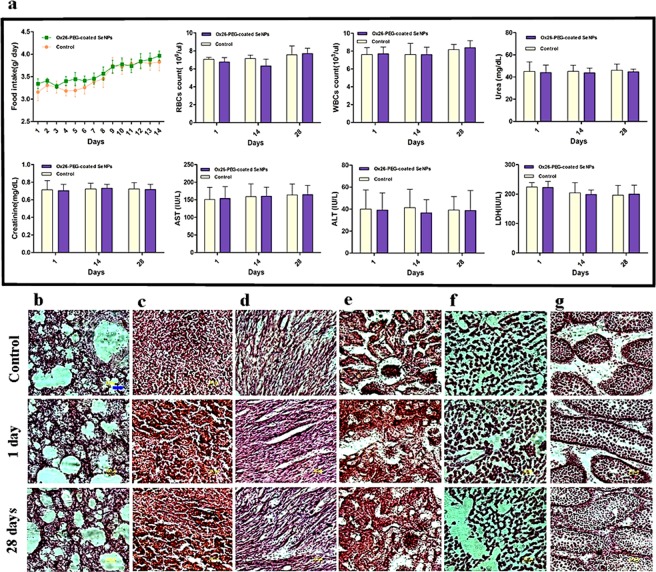
Study of potential adverse effects of Se of intraperitoneally administrated OX26-PEG SeNPs at dose of 1000 μg/mL by examining (**a**) daily food intake, haematological parameters (RBC and WBC levels) and biochemical factors (urea, creatinine, AST, ALT, LDH levels). No significant variations were observed for the groups exposed to OX26-PEG-Se NPs as compared to control ones after 1, 14 and 28 days (data expressed as ± s.d and N = 6 animals per group). Histopathological analysis of lung (**b**), spleen (**c**), heart (**d**), kidney (**e**), liver (**f**) and testis (**g**) in control, 1 day and 28 days after intraperitoneal administration of the NPs did not show any cell and/or tissue damages (the scale bar is 100 μm; blue line).

NPs may have adverse effects; we investigated the emergence of systemic toxicity. First, we did not observe any change in the daily food intake due to NP administration (Fig. 4a). Second, we performed haematological analysis after administration of the OX26-PEG-Se NPs (1000 μg/mL at 1, 14 and 28 days) and evaluated red and white blood cells count and other haematological parameters (Fig. S7). Again, no significant differences were observed between the OX26-PEGSe NP and control groups. Third, we analyzed renal function because of commonly-observed significant uptake of nanoparticles by kidney. No significant increases in urea and creatinine levels were observed for the OX26-PEG-Se NP group as compared to the control one (Fig. 4a). Moreover, in order to study whether the OX26-PEG-Se NPs may involve in detrimental effects on liver, we measured the AST and ALT serum levels, as important indicators of the hepatic injury. Again, we did not observe a significant difference between the OX26-PEG-Se NPs and control groups (Fig. 4a). We also studied the effects of the OX26-PEG Se NPs on other organs, for which the serum LDH level and morphology of different tissues were evaluated. In this regard, the histopathological analysis of the heart, spleen, liver, kidney, lung and testis (in fact, our previous study showed that testis is one of the organs with high uptake of NPs)^37^ showed no observable morphological damages after the OX26-PEG-Se NPs administration (either one day or 28 days after the injection (Fig. 4b–g for morphological aspects). These results demonstrated that the functionalized Se NPs can behave as biocompatible nanomaterials, without any Se-related side effects at the desired dose of 1000 μg/mL, for *in vivo* therapeutic applications.

### Molecular targeting of OX26-PEG-Se NPs

In order to gain mechanistic insights into the observed therapeutic effect of the Se NPs, we studied pathways related to metabolism, oxidative stress and inflammatory response.

#### Targeting molecular pathways involved in cellular metabolic activity

**Figure 5:**
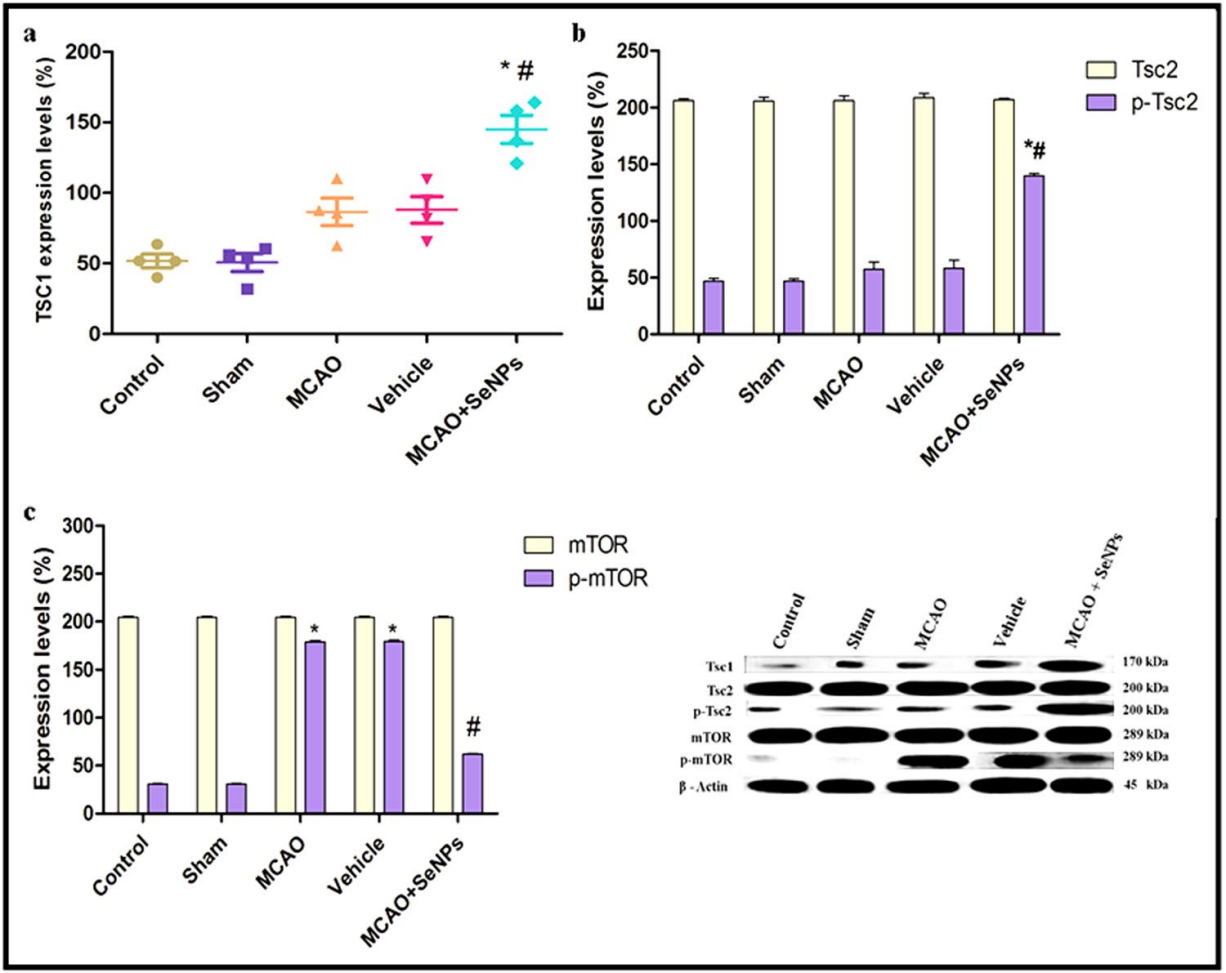
Western extracted data shows expression levels of (**a**) Tsc1, (**b**) Tsc2 and p-Tsc2, and (**c**) mTOR and p-mTOR proteins in Wistar rats treated by MCAO + Se NPs as compared to other groups (*p < 0.001 compared to control and sham, ^#^p < 0.01 compared to MCAO and vehicle, N = 4 animals per group).

The mammalian target of rapamycin (mTOR) is a key molecule that integrates multiple extracellular and intracellular signals to regulate cell survival. It has been shown that mTOR controls cellular energy consumption and its activity is altered in pathological conditions such is ischemic and oxidative stress^38^. In this regard, the impact of the OX26-PEG-Se NPs on targeting mTOR signaling pathways was studied by monitoring the activity of the Tsc1/Tsc2 complex, central negative regulator of mTORC1, and expression of Tsc1 and phosphorylation levels of Tsc2 proteins. The Tsc1 protein induces protective autophagy and neuroprotection against focal cerebral ischemia via Tsc2 phosphorylation and subsequently mTORC1 suppression. The Tsc2 phosphorylation decreases mTORC1 activity through direct inhibition of the small GTPase Rheb (a positive regulator of mTORC1 complex)^39^. Here, the Tsc1 expression was found significantly higher in rat hippocampal cells exposed to the functionalized Se NPs + MCAO as compared to other groups (Fig. 5a). Likewise, to confirm whether the Tsc1 expression alters neuronal fate via an mTORC1-dependent mechanism, we determined the Tsc2 phosphorylation in rat hippocampal neurons. The total content of Tsc2 protein was not changed in different groups, whereas the Tsc2 phosphorylation was found higher in the functionalized Se NPs + MCAO as compared to other groups, indicating suppression of the mTORC1 activity (Fig. 5b). To find out the link between neuronal survival and mTOR signaling, we measured total mTOR and phosphorylated-mTOR (p-mTOR) expressions in different groups. Expression of the total mTOR protein was not affected by MCAO or Se NPs + MCAO treatments. However, increased p-mTOR expression was detected in the MCAO and vehicle groups. Interestingly, OX26 PEG Se NPs treatment before MCAO significantly decreased p-mTOR expression (Fig. 5c). This is associated with the neuroprotective effects of the OX26-PEG-Se NPs in suppression of mTORC1 complex.

#### Targeting molecular pathways involved in controlling antioxidant defense system

**Figure 6:**
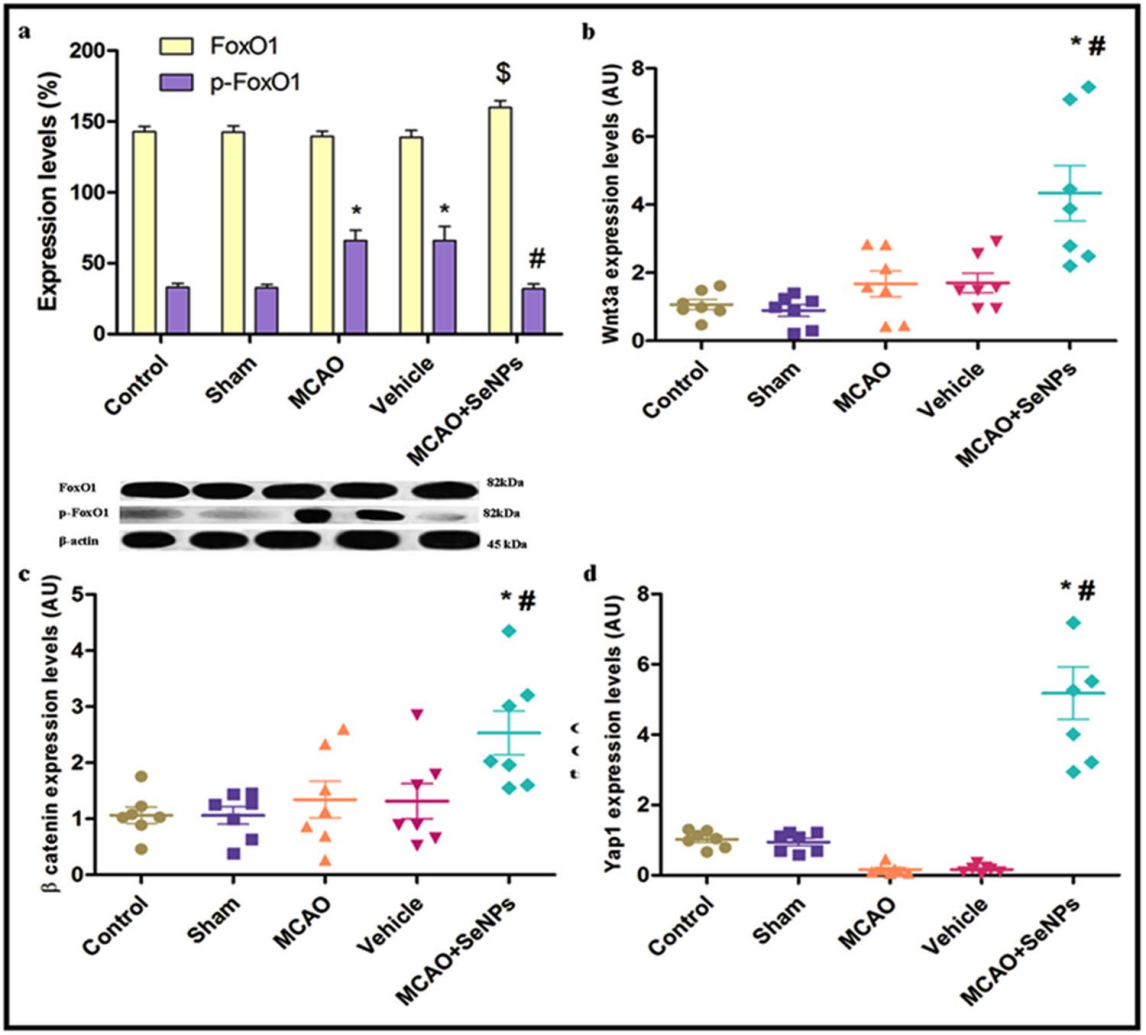
Western extracted data shows expression levels of (**a**) cytoplasmic FoxO1 and p-FoxO1 proteins in Wistar rats treated by MCAO + Se NPs as compared to other groups (*p < 0.001 compared to control and sham, ^#^p < 0.01 compared to MCAO and vehicle, $ p < 0.05 for MCAO + Se NP group as compared to other groups, N = 4 animals per group). RT-PCR extracted data shows expression levels of the mRNA level of (**b**) Wnt3a (*p < 0.001 compared to control and sham, and ^#^p < 0.01 compared to MCAO and vehicle), c) β-catenin (*p < 0.01 compared to control and sham, and ^#^p < 0.05 compared to MCAO and vehicle), d) Yap1 (*p < 0.001 compared to control and sham, ^#^p < 0.001 compared to MCAO and vehicle).

We also tried to decipher the link between total and phosphorylated FoxO1 protein and neuronal survival in different groups. FoxO molecular targets include different genes coding for both intra and extracellular antioxidant proteins. FoxO1 increases transcription of selenoenzymes including glutathione peroxidase 1 (GPx1), and other antioxidant enzymes such as superoxide dismutase (SOD) and catalase. Stress-responsive kinases like Akt and ERK catalyze the FoxO1 phosphorylating that leads to its inactivation and nuclear exclusion^40^. OX26-PEG-Se NPs treatment before MCAO increased the expression of the total FoxO1 protein, while it dramatically decreased the expression of the p-FoxO1 protein, suggesting promotion of endogenous antioxidant defenses in rat hippocampal cells (Fig. 6a). Cross-talk between FoxO and β-catenin/Wnt pathways has been reported in previous studies. For example, activated Wnt inhibits the β-catenin degradation that leads to an increase on cytosolic β–catenin protein. Cytosolic β–catenin was transferred to the nucleus and interacts with FoxO proteins to promote the expression of the antioxidant enzymes such as Mn-SOD^41^. To examine whether the OX26-PEG-Se NPs may target Wnt/β-catenin cellular signaling pathway to induce neuroprotection against oxidative stress was studied in next step. 24 h after the MCAO induction, no significant increase in mRNA level of Wnt3a was observed in rat hippocampal tissue as compared to control and sham groups. OX26-PEG-Se NPs treatment before MCAO led to a significant increase on mRNA level of Wnt3a in hippocampal tissue (Fig. 6b). OX26 PEG Se.

NPs also increased β-catenin mRNA at hippocampal tissue 24 h after MCAO (Fig. 6c). These results strongly suggested that the OX26-PEG-Se NPs reinforce the endogenous antioxidant defense by targeting parallel signaling pathways. It has been shown that Repressor element 1 silencing transcription factor (REST) overexpression by neurotoxic stimuli confers neuronal survival via a Wnt-dependent mechanism^9^. One of the components of the hippo signaling pathway (Yap1), may directly bind to FoxO1 and promotes its transcriptional activity from antioxidant enzymes. The Yap1 down regulation increases the vulnerability of the cells to reactive oxygen species (ROS), leading to neuronal cell death^42^. Confirming the abovementioned data, we found that the Yap1mRNA level decreased by MCAO induction. Conversely, the mRNA level of Yap1 dramatically increased relative to other groups, 24 h after MCAO in OX26-PEG-Se NPs treated group (Fig. 6d). A previous study reported that pretreatment with 1 mg/kg of rosuvastatin (present in phase II of therapies for acute ischemic stroke in clinical research) once a day from 7 days before induction of MCAO decreased I/R injury following ischemic stroke through attenuation of oxidative stress^43^. Likewise, our finding showed that pretreatment with 2 mg/kg (single dose) of the OX26-PEG-Se NPs could activate involved in controlling antioxidant defense system.

#### Targeting molecular pathways involved in protein quality control

**Figure 7:**
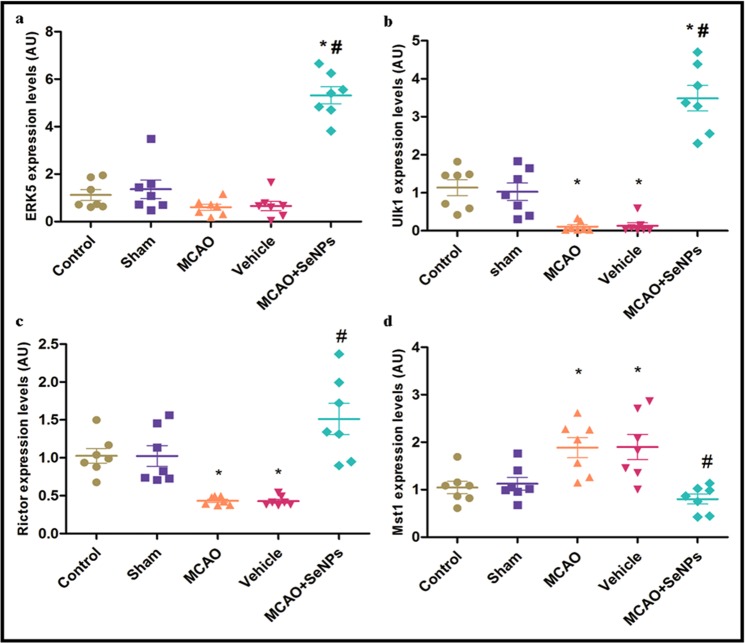
(**a,b**) Intraperitoneal administration of OX26-PEG-Se NPs significantly increased mRNA level of ERK5 and ULK1 gene (*p < 0.001 compared to control and sham, ^#^p < 0.001 compared to MCAO and vehicle). (**c**) mRNA level of Rictor significantly decreased in MCAO and vehicle compared to control and sham (*p < 0.05). OX26-PEG-Se NPs increased mRNA level ofRictor compared to MCAO and vehicle (^#^p < 0.001). (**d**) Mst1 exhibited an increased mRNA level in MCAO and vehicle relative to control and sham (*p < 0.05). Treatment with OX26-PEG-Se NPs significantly decreased mRNA level of Mst1 compared to MCAO and vehicle (^#^p < 0.01, N = 7 animals per group).

In the ischemic brain tissue activated mTORC1 also suppresses the activity of the ubiquitin-proteasome system (UPS) that leads to accumulation of the misfolded and damaged proteins, and consequently, neuronal cell death. The p-mTORC1 can inhibit the activity of ERK5 gene acting as the central positive regulator of UPS in mammals^44^. Hence, the effect of OX26-PEG-Se NPs on the USP function in hippocampal cells after MCAO was investigated. Induction of the MCAO slightly decreased the mRNA level of ERK5 gene, whereas the OX26-PEG-Se NPs significantly increased ERK5 mRNA. This suggested the capability of the OX26-PEG-Se NPs in preserving neuronal cell survival through restoration of UPS activity (Fig. 7a). On the other hand, it is well documented that autophagy is a collaborator of UPS in neuroprotection via degradation of specific protein aggregates and damaged organelles in neurons. Indeed, UPS mediated degradation of small and soluble aggregated proteins due to the 26S proteasome and the pore-size limitations of the retrotranslocation channel whereas large misfolded proteins and damaged organelles move to autophagy^45^. Previous evidences have showed that excessive activity of mTORC1 activity results in impairment of mitochondrial electron transport chain, overgeneration of ROS, suppression of autophagy, and subsequent poor cell survival^46^. On the other hand, accumulating evidences showed that FoxO1 activation regulates autophagy in neurons^47^. Hence, a key question is whether the OX26-PEG-Se NPs contributes to neuronal survival by removing damaged mitochondria and large misfolded proteins through an autophagy-dependent mechanism. To clarify this issue, we measured mRNA level of ULK1 gene in rat hippocampal tissue by RT PCR assay. The RT-PCR analysis showed a decrease in the ULK1 mRNA level 24 h after reperfusion. OX26-PEG-Se NPs administration before MCAO significantly increased the ULK1 mRNA level, suggesting protective autophagy induction (Fig. 7b).

#### Targeting molecular pathways involved in memory consolidation by hippocampal neurons

Cognitive and behavioral outcome of the OX26-PEG-Se NPs treatment after cerebral ischemia was evaluated by studying its effects on mTORC2 complex at molecular levels. mTORC2 complex plays a major role in the conversion from short- to long-term memory and consolidation of long-term memory through regulating of actin polymerization^48^. Here, the impact of OX26-PEG-Se NPs on restoring the rictor (core subunit of mTORC2 complex) mRNA at hippocampal neurons and preserving the cognitive function in rats suffered focal cerebral ischemia was examined. Rictor mRNA level was decreased in hippocampal tissues prepared from MCAO and vehicle groups. In contrast, the rictor mRNA level significantly increased in hippocampal tissues treated by the OX26-PEG-Se NPs, indicating recovery of cognitive functions and increase of the activity of mTORC2 (Fig. 7c). It has been reported that Donepezil, a clinical neuroprotective drug, attenuates the memory deficit at 5 mg/kg dose for 21 consecutive days following a period of cerebral ischemia for 10 minute^49^. Compared with Donepezil that is in phase II of therapies for acute ischemic stroke, this study showed that a short-term therapy (single administration) of OX26-PEG-Se NPs with lower dose could activate molecular pathways involved in memory consolidation by hippocampal neurons.

#### Targeting molecular pathways involved in neuronal cell apoptosis

A tight association between mTORC2 and hippo signaling pathways has been reported by previous studies. For instance, rictor/mTORC2 decreases the extent of the cell loss through Mst1 inhibition^50^. Mst1 (mammalian Ste20-like kinase 1) is a pro-apoptotic kinase and a component of the hippo signaling pathway that increases susceptibility of neurons to the apoptotic cell death by improving the interaction between Beclin1 and Bcl-2, and subsequently, suppression of the autophagy^51^. Here, to evaluate the role of Mst1 in the vulnerability of hippocampal neurons, we also assayed mRNA level of Mst1 gene. The RT-PCR assay revealed that mRNA level of Mst1 significantly increased following MCAO induction. OX26-PEG-Se NPs administration before MCAO decreased the Mst1 mRNA level in hippocampal tissue as compared to the MCAO and vehicle groups.

**Figure 8:**
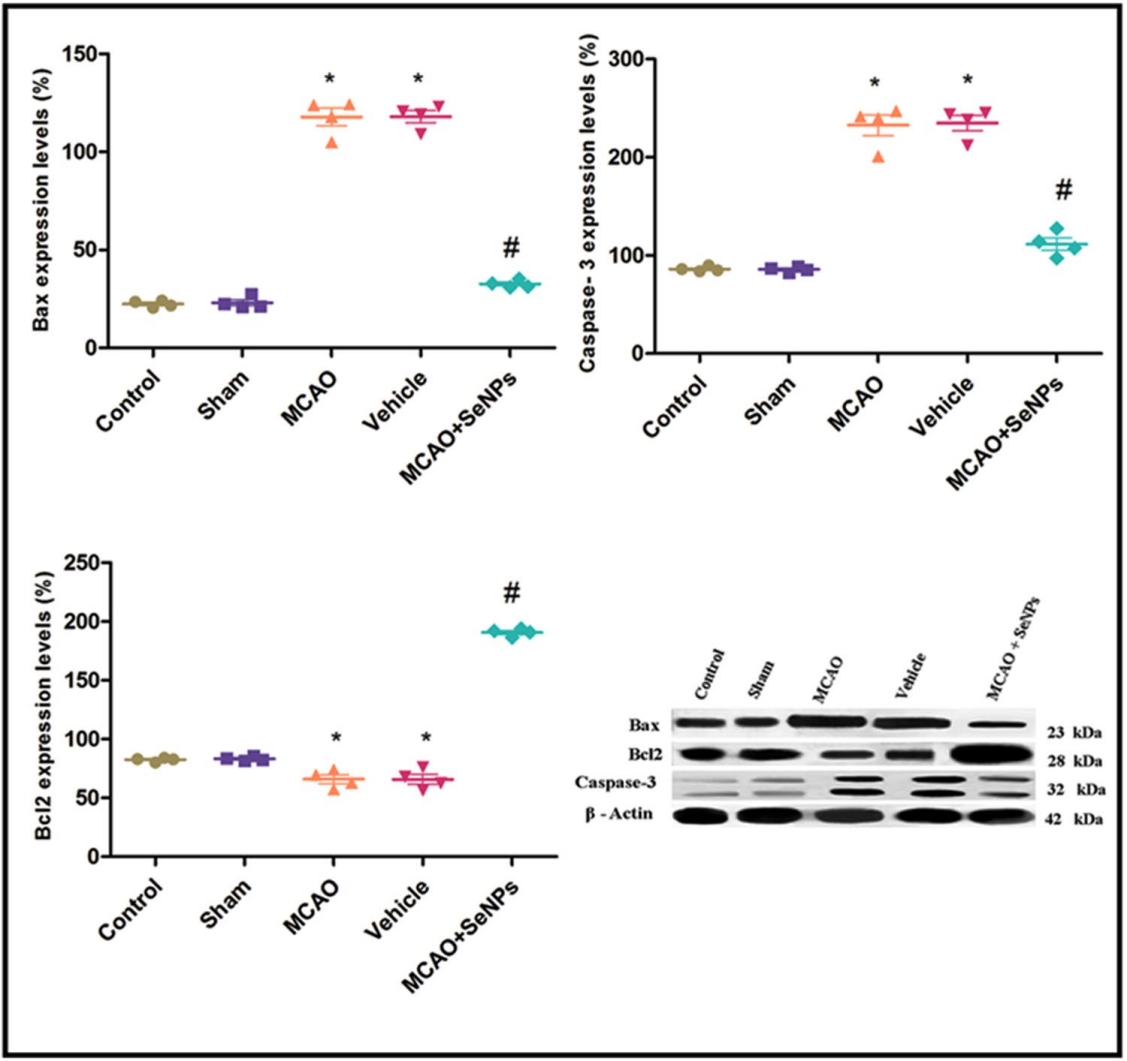
The expression level of (**a**) Bax, (**b**) Caspase3 proteins and (**c**) Bcl2 24 h after reperfusion. Intraperitoneal administration of OX26-PEG-Se NPs could maintain the low level of Bax and cleaved caspase-3 proapoptic factors under oxidative stress (*p < 0.01 compared to control and sham, ^#^p < 0.001 compared to MCAO and vehicle, N = 4 animals per group).

To confirm the anti-apoptotic property of the OX26-PEG-Se NPs in rat hippocampal neurons against ischemia, we assessed the expression of pro-apoptotic and anti-apoptotic proteins by Western blot assay. Increased expression of the pro-apoptotic Bax and cleaved caspase 3 proteins in MCAO group was reversed by OX26-PEG-Se NPs administration, indicating the limited apoptotic cell death by functionalized Se NPs (Fig. 8a–c). In parallel, decreased expression of the anti-apoptotic protein Bcl2 in MCAO group, significantly reversed by OX26-PEG-Se NPs treatment, confirming the anti-apoptotic effects of the OX26-PEG-Se NPs. Above mentioned results show that targeted therapy with OX26-PEG-Se NPs can act similar or better than antiplatelet clinical drugs such as aspirin. For instance, aspirin indicated a significantly lower degree of apoptosis in MCAO rats at 100 mg/kg concentration whereas this study showed that OX26-PEG-Se NPs administration at 1000 ug/ml (2 mg/kg) concentration markedly decreased apoptosis^52^. Likewise, treatment with OX26-PEG-Se NPs at 2 mg/kg dose could decrease apoptotic cell death similar to Edaravone, a free radical scavenger drug, at 3 mg/kg dose that is in phase II of therapies for acute ischemic stroke^53^.

#### Targeting molecular pathways involved in inflammatory responses after stroke

**Figure 9:**
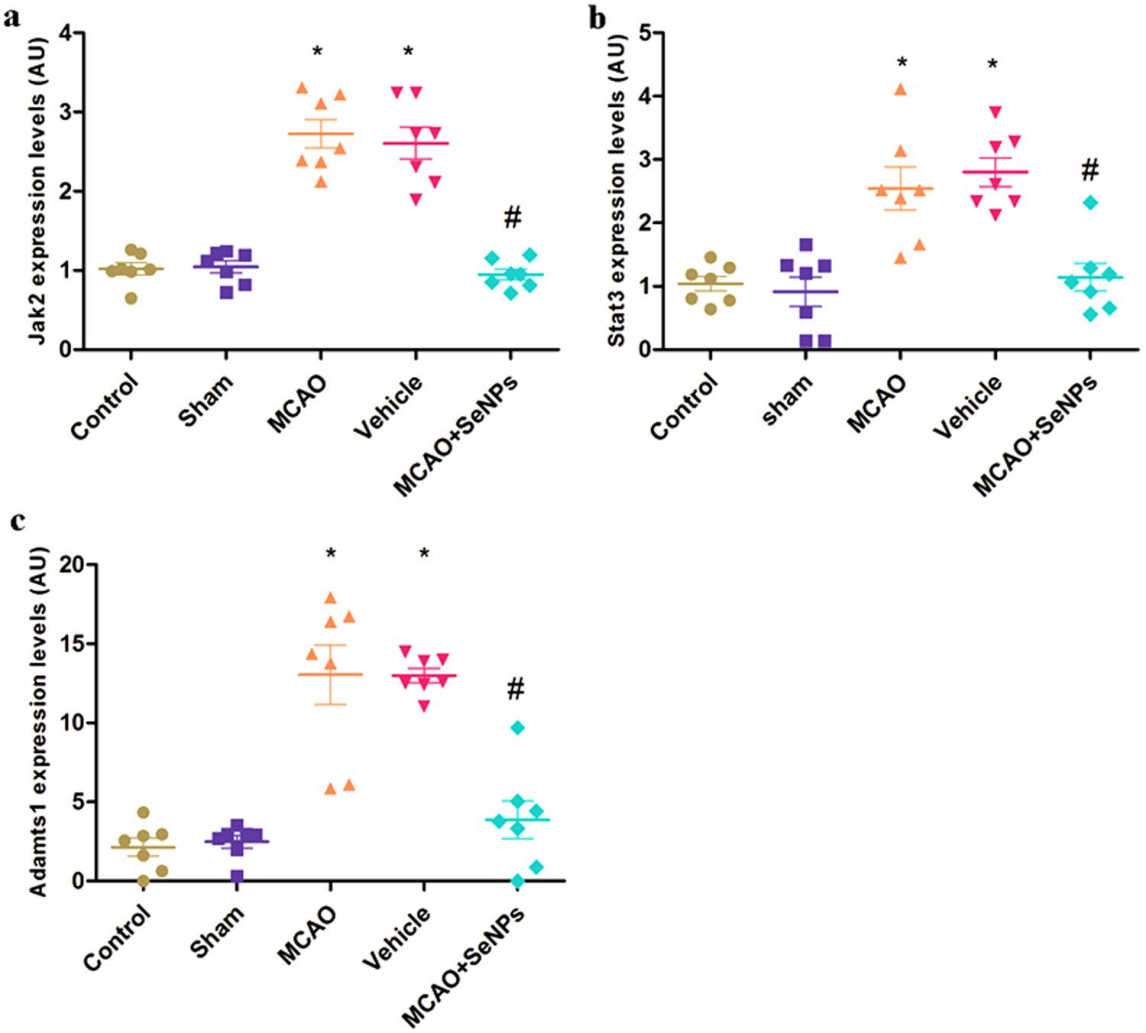
The mRNA level of (**a**) Jak2 and (**b**) Stat3 (*p < 0.001 compared to control and sham and ^#^p < 0.001 compared to MCAO and vehicle), and (**c**) Adamts-1 (*p < 0.001 compared to control and sham, and ^#^p < 0.001 compared to MCAO and vehicle) genes of Wistar rats suffered focal cerebral ischemia, 24 h after treating by OX26-PEG-Se NPs (for all tests, N = 7 animals per group).

The jak2/stat3 pathway plays an important role in various diseases^54^. Focal cerebral ischemia is often accompanied by activation of macrophages/microglia, leading to acute inflammatory responses and neuronal damage through aberrant activation of the jak2/stat3 pathway^55^. Hence, we also investigated the anti-inflammatory effect of the OX26-PEG-Se NPs by targeting Jak2/Stat3 pathways. Using RT-PCR assay, it was found that the MCAO induction led to a significant increase in Jak2 mRNA level 24 h after ischemic insult. The expression of Jak2 was markedly suppressed in presence of the OX26-PEG-Se NPs (Fig. 9a). We further investigated Stat3 gene expression in different experimental groups. Our results showed relatively increased expression of the Stat3 in MCAO and vehicle groups, which have been reversed byOX26-PEG-Se NPs treatment, indicating functionalized Se NPs potential on attenuating the inflammatory reactions (Fig. 9b). Furthermore, after stroke aberrant expression of some members of the Adamts family such as Adamts-1 and -4 by inflammatory cells leads to the extracellular matrix (ECM) breakdown followed by neural cell death^56^. To determine if the OX26-PEG-Se NPs might contribute to maintaining the structural integrity of ECM in rat hippocampal tissue, we measured Adamts-1 mRNA levels in all groups. The Adamts-1 mRNA level was elevated by MCAO in the first 24 h after ischemic insult, that it significantly decreased by OX26-PEG-Se NPs treatment (Fig. 9c). These findings indicate that the OX26-PEG-Se NPs also protected the ECM integrity that plays critical role on preserving neural cell survival during and after stroke. Compared with some neuroprotective drugs such as statins that are in phase I therapies for acute ischemic stroke and contribute to reduction of neuroinflammation following stroke, OX26-PEG-Se NPs showed similar action with lower dose^57^. Although Tissue plasminogen activator (tPA) is considered the gold standard treatment for ischemic stroke, one of its side effects is enhancement of inflammatory response in brain capillaries and subsequently neuronal cell damage after stroke^58^. Compared with tPA, this study showed that the OX26-PEG-Se NPs could inhibit molecular pathways involved in inflammatory responses after stroke.

## Conclusion

**Figure 10:**
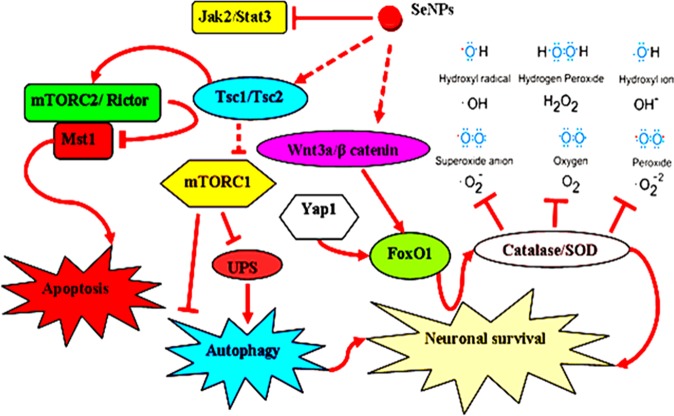
Proposed signaling pathways involved in SeNPs-induced protection.

Collectively, we successfully synthesized a biodegradable nanoparticulate system capable of targeting the brain for stroke therapy. OX26 antibody surface decoration remarkably enhanced the targeted transport of Se NPs to the brain through transferrin receptor-mediated endocytosis. Our *in vitro* analysis revealed that the major localization of the NPs was in the nucleus. More importantly, we found that Se NPs contributed to neuronal survival by targeting different cellular signaling pathways (Fig. 10) that regulate cellular metabolic state (TSC1/TSC2, p-mTOR, mTORC1), oxidative defense system (FoxO1, β-catenin/Wnt, Yap1), inflammatory reactions (jak2/stat3, Adamts-1), autophagy and apoptotic cell death (Mst1, ULK1, Bax, Caspase-3 and Bcl-2), as well as functional properties of the hippocampal neurons (rictor/mTORC2).

## Methods and Materials

### Preparation of OX26-PEG-coated Se NPs

Here, we describe our synthesis protocol for preparation of selenium nanoparticles (NPs) with selenium core and polyethylene glycol (PEG) surface coating. Initially, 2 μg/mL poly (ethylene glycol)-carboxylic acid functionalized (average Mw 5 kDa, Sigma- Aldrich) was directly mixed with 1800 μL of 0.1 M selenious acid 98% (Sigma-Aldrich) in 10 mL deionized (DI) water, by constant stirring. Then, 3 mL of aqueous 0.1 M ascorbic acid solution (A92902, Sigma-Aldrich) was added dropwise and the resulting solution was stirred at room temperature. After 30 min, 20 μL EDC/NHS (Sigma-Aldrich) (75 mM/30 mM, v/v, 1:1) solutions was directly added into the Se-containing solution and the mixture was stirred for 30 min. After washing by DI water, 5 μL of CD71 antibody (OX26) (Santa Cruz Biotechnology) was added into Se suspension and the mixture was stirred at room temperature for 5 h. The non-bonded OX26 antibodies were removed by centrifugation at 20,000 rpm for 60 min at 4 °C. Then, the pellets were resuspended in Milli-Q water. To evaluate the impact of NP-protein interactions on cellular uptake and survival *in vitro*, FITC (Fluorescein isothiocyanate) - OX26 (Santa Cruz Biotechnology) was used instead of CD71 antibody (OX26).

### Material characterizations

The size distribution and zeta potential of the OX26-PEG-Se NPs were evaluated by a Nano-ZS instrument (Malvern Zeta sizer Nano ZS90, Worcestershire, UK) in water, phosphate buffered, human plasma, and the complete cell culture medium (cMEM) consisting of MEM (supplemented with 10% fetal calf serum (Gibco), 1% penicillin/streptomycin (Gibco) at 25 °C and 37 °C. Particle morphology and localization were determined by transmission electron microscopy (TEM, H-7650) at an accelerating voltage of 80 KV. For evaluation of morphology, a droplet of diluted nanoparticles was dropped onto carbon-coated copper grids, (some of them were also prepared by using a desktop sputtering system (Nanostructured Coating Co., Iran). Then, the samples were dried at room temperature for two days. To evaluate neuronal localization of OX26-PEG-Se NPs, differentiated PC12 cells were exposed to the NPs (with sizes ~25 nm and concentration of ~100 μg/mL) at 37 °C for 1 h and 3 h. The RPMI media was removed and the cells were washed twice with PBS buffer and harvested with trypsin/EDTA for 4 min. Then, the cells were fixed at room temperature in 2.5% glutaraldehyde in 0.1 M Sorensen phosphate buffer (pH 7.4) for 2 h and washed with buffer (pH 7.3). Then, 1% osmium tetroxide (in DI water) was used to postfix the samples for 2 h. After that, the cells were dehydrated in increasing concentrations of ethanol (from 70% up to 100%), and transferred to copper grids following several sequential steps of (i) immersion in an ethanol/Epon (1:1 vol/vol) mixture for 1 h, (ii) transferring and embedding in pure Epon at 37 °C for 2 h, (iii) polymerization at 40 °C for 72 h and (iv) preparation of ultrathin sections of 100 nm thickness by ultramicrotome. Then, the cells were stained with uranyl acetate and lead citrate.

### Quantitation of OX26 antibody Conjugated to SeNPs

Bradford assay was used to evaluate the number of immobilized OX26 antibody molecules onto the surface of SeNPs. In brief, standard solutions of OX26 antibody (1, 5 and 10 μg/mL) were provided in 2 mM borate buffer (pH 8.0). The standard solutions and OX26 conjugated nanoparticles were centrifuged at 5000 g for 5 minutes. Then, 90 μL of the standard solutions or the supernatant was transferred to a 96-well plate. After dilution of each sample and standard with 70 μL of 2 mM borate buffer, 40 μL of the Bio-Rad reagent was added to each well. After 15 minutes incubation at room temperature, UV−visible absorption was determined at 595 nm to quantify OX26 antibody in the supernatant. Finally, total number of immobilized OX26 antibody molecules onto the surface of SeNPs was calculated as the difference between the number of OX26 antibody molecules added to the SeNP suspension and antibody remaining in the supernatant.

### Cell culture

In order to obtain the required cell density, cells were counted before each experiment. PC12 cells (original batches from Pasteur Institute of Iran), were isolated from a transplantable rat adrenal pheochromocytoma. In order to study neurobiological mechanisms, PC12 cells were cultured in RPMI 1640 medium supplemented with 10% fetal calf serum (Gibco), 1% penicillin/streptomycin at 37 °C in a humidified 5% CO_2_ atmosphere. To induce neuronal differentiation, cells were treated with NGF and 1% serum (1:100) for 5 days (50). The human MCF7 breast adenocarcinoma cells (original batches from Pasteur Institute of Iran) were maintained in Dulbecco’s modified Eagle’s medium (DMEM) supplemented with 10% fetal bovine serum and 1% penicillin/streptomycin at 37 °C in a humidified incubator with 5% CO_2_ atmosphere.

### Oxygen glucose deprivation

The OGD was induced in human MCF7 cells and rat pheochromocytoma (PC12) cells (under differentiation state) through immersion in 500 μL deoxygenated custom neurobasal medium without glucose, aspartate, glutamate, glutamine or pyruvate (Invitrogen). Oxygen was removed the immersion solution by a premixed gas (85% N_2_, 10% H_2_, 5% CO_2_) within 30 min. Following OGD, both human MCF7 cells and rat PC12 cells were randomized into two groups (SF and cMEM) and incubated with 25 μg/mL FITC-OX26-PEG-coated SeNPs in oxygen-glucose deprivation/reperfusion condition at different times. Fluorescence microscope was used to evaluate cellular uptake and survival at different times.

### Flow cytometry

To do this, 3 × 10^5^ cells were resuspended in a given volume of cMEM and into individual cell-culture-flask (25 cm). 5flasks of differentiated PC12 cells were exposure to FITCPEG-coated Se NPs for given times. To evaluate the impact of OX26-PEG-coated Se NPs on targeting capability by flow cytometry, after exposing the NPs to the cells for the required times, the NP suspension was removed, then the cells were washed twice with PBS buffer and harvested with trypsin/EDTA for 4 min. Formaline 4% solution (Sigma) was used to fix cells for 20 min. Finally, the cells were resuspended in PBS buffer for flow cytometry assay. In parallel, 5 flasks of differentiated PC12 cells were also incubated with FITC-OX26-PEG-coated Se NPs, with the same experimental conditions.

### Fluorescence microscopy

For qualitative analysis of the impact of NP-protein interactions on cellular uptake and survival, MCF7 cells were allowed to attach for 24 h as described above (in DMEM supplemented with 10% FBS). Then the medium was removed from the cell-culture-flask and after washing the cells three times with cold PBS buffer, an individual incubation of FITC-OX26-PEG-coated Se NPs in SF and the cMEM was performed for different periods of time. Then, the cells were washed three times with cold PBS buffer to remove the OX26-PEG-coated Se NPs from outside of the cells. Cellular uptake and survival were then investigated by fluorescence microscope (Nikon Eclipse 230 80i).

### ICP-OES analysis

This analysis was used to determine the Se content in different tissues of male Wistar rats after intraperitoneal administration of PEG-coated Se NPs and Ox26-PEG-coated SeNPs after 24 h exposure. The brain, liver and kidney tissue samples were collected, weighed and homogenized for 5 min in 3 mL cold PBS with a tissue homogenizer. Then, samples were digested with nitric acid and hydrogen peroxide (4:1, v/v) at 70 °C for 6 min. The samples were cooled at room temperature and transferred to 15 mL ICP-OES tubes. All samples were analyzed in triplicate.

### Ethical approval

The present study was conducted in strict accordance with the Guide for the Care and Use of Laboratory Animals published by National Research Council of the National Academies, USA (2011) and was approved by the Institutional Animal Ethical Committee of Iran University of Medical Science. Nine weeks old Male Wistar rats weighing 250–300 g were purchased from the Center for Experimental Animals of Iran University of Medical Science. Animals were kept in the temperature controlled room under a 12/12 h light/dark with unlimited access to food and water. To maintain social interaction, rats were placed in four per cage. They were randomly subdivided into 5 groups: a control group of healthy mice (normal, n = 12), sham operated control group (sham, n = 12), MCAO group (MCAO, n = 12), OX26-PEGantibodygroup (Vehicle, n = 12), MCAO-Se NPs group (treatment, n = 12).

### MCAO model

Rats were anesthetized with intraperitoneal injection of a mixture of ketamine and xylazine. The right common carotid artery (CCA) was exposed via a midline neck incision; then after isolation of external carotid artery (ECA) and internal carotid artery (ICA), ECA was tied. To occlude the MCA, a 4–0 monofilament nylon suture was inserted from the right CCA to the ICA. After 30 min, monofilament was gently removed to allow recirculation of cerebral blood flow for 22 h. All above procedures were repeated for sham-operated animals except occlusion of the MCA. Intraperitoneal administration (1000ug/ml) of OX26-PEG-coated Se NPs was performed 1 h before ischemic stroke.

### Tissue preparation

Rats were sacrificed under deep anesthesia with a mixture of ketamine/xylazine (final dose: 30/10 mg/kg body weight, IP). The rat brains were removed; then the ipsilateral ischemic hippocampus was quickly dissected from the brain tissue and saved at −80 °C prior to RT-PCR and Western blot assays.

### Real time RT–PCR

Real-time PCR was run for 45 cycles. Single peak melting curves were described as determinants of purity. In order to extract RNA, frozen hippocampus tissue was powdered in a mortar and pestle by the addition of liquid nitrogen; then homogenized in TRIzol (Invitrogen) on ice for 5 minute. The samples were incubated with 250 mL chloroform (Sigma-Aldrich) for 3–5 min at room temperature and centrifuged at 13,000 g for 20 min at 4 °C. Afterward, the upper aqueous phase containing the RNA was separated and incubated with an equal volume of cold isopropanol (Sigma-Aldrich) overnight at −20 °C, followed by centrifuging at 13,000 g for 11 min at 4 °C. Finally, the pellets were mixed with 75% ethanol and centrifuged at 7,700 g for 6 min at 4 °C. Ethanol was removed and pellet dissolved in 25 μL of DEPC-treated H2O. The RNA was stored at −80 °C prior to cDNA synthesis. The RNA purity and concentration were determined at 260/280 nm by using a Nanodrop spectrophotometer (NANO DRAP oNEC, Thermo Scientific). The RNA series were converted to cDNA ones by a Dart cDNA kit (EURx Company, Poland) according to the manufacturer’s protocol. Real-time PCR was performed using SYBR®.

Premix Ex Taq ™ II (TliRNaseH Plus, RR820Q) (TAKARA). The detection of PCR products was based on the DNA-binding dye SYBR Green. Temperature cycling was 94 °C for 30 s, 56 °C for 30 s, and 68 °C for 1 min and repeated for 45 cycles. 2 − ΔΔCT method was used to calculate relative changes in gene expression. Results were reported as arbitrary units. Serial dilution of titrated product was performed to generate a standard curve for each primer pair. Internal control used for this study was GAPDH. Primers used in present study are listed in Table S6.

### Western blot assay

Frozen hippocampus tissue was homogenized in RIPA buffer (protease and phosphatase inhibitor) (Sigma-Aldrich). After a sonication step, right hippocampus lysate was subjected to centrifugation at 13300 g at 4 °C for 20 min. The supernatant was removed and protein concentration was determined using the Nanodrop equipment. Then, 4–20% gradient SDS/PAGE (sodium dodecyl sulfate polyacrylamide gel electrophoresis) was used to separate soluble protein (50 μg). Separated soluble proteins were transferred onto PVDF (Immobilon®-FL PVDF membrane pore size 0.45 μm-Sigma-Aldrich). To evaluate the loading, PVDF membranes were stained with Sypro or Ponceau S. Then, nonspecific reaction sites were blocked with a blocking solution (3% solution of BSA and 0.1% Tween-20 (TBST)). The antibodies used for this experiment are listed in Table S7. After incubation with primary antibodies, PVDF membranes were exposed to diluted secondary antibody in blocking buffer for 1 h at room temperature. Then, chemiluminescent HRP Substrate (Millipore) was served as detector of immunoreactivity onto Kodak X-OMAT films for 5 min. β-Actin served as loading control. To evaluate the mean values of proteins, ratio of expression of each protein to the expression of the β-actin in the same sample was determined by Alpha Ease ® FC Imaging System based on their optical densities.

### Statistical analysis

Experiments were carried out in duplicates or triplicates and the results are shown as Mean ± standard deviation (SD). The statistical analysis of data was performed using prism 5. To analyze the difference between two groups, two-tailed Student’s t-test was served and the differences between three or more groups were performed by one-way ANOVA. Values of P < 0.05 were considered to denote statistical significance in all cases.

## Supplementary information

Supplementary information
