# Supplementary material for: Selenium nanoparticles for targeted stroke therapy through modulation of inflammatory and metabolic signaling
Source: Sci Rep. 2019 Apr 15;9:6044. doi: 10.1038/s41598-019-42633-9 (PMC6465364; doi:10.1038/s41598-019-42633-9)
Supplement: Supplementary file 1 — Supplementary information [file 41598_2019_42633_MOESM1_ESM.docx]

**Supporting Information**

**Selenium nanoparticles for targeted stroke therapy through modulation of inflammatory and metabolic signaling**

**Hamed Amani^1^, Rouhollah Habibey^2^, Fereshteh Shokri^3^,** **Seyed Javad Hajmiresmail^4^, Omid Akhavan^5^ *, Alireza Mashaghi^6,7^ *, Hamidreza Pazoki-Toroudi^8^***

^1^ Department of medical nanotechnology, Faculty of Advanced Technologies in Medicine, Iran University of Medical Science, Iran

^2^ Department of Neuroscience and Brain Technologies-Istituto Italiano di Technologia, Via Morego, Italy

^3^ International Pharmaceutical Federation, The Hague, Netherlands

^4^ Department of Cardiology, Iran University of Medical Sciences, Tehran, Iran

^5^ Department of Physics, Sharif University of Technology, Iran

^6^ Leiden Academic Centre for Drug Research, Faculty of Science, Leiden University, Netherlands

^7^ Harvard Medical School, Harvard University, Boston, USA

^8^ Physiology Research Center and Department of Physiology, Faculty of Medicine, Iran University of Medical Sciences, Iran

*Corresponding authors: a.mashaghi.tabari@lacdr.leidenuniv.nl; [oakhavan@sharif.edu](mailto:oakhavan@sharif.edu); pazoki.h@iums.ac.ir

**Supplementary Figures**


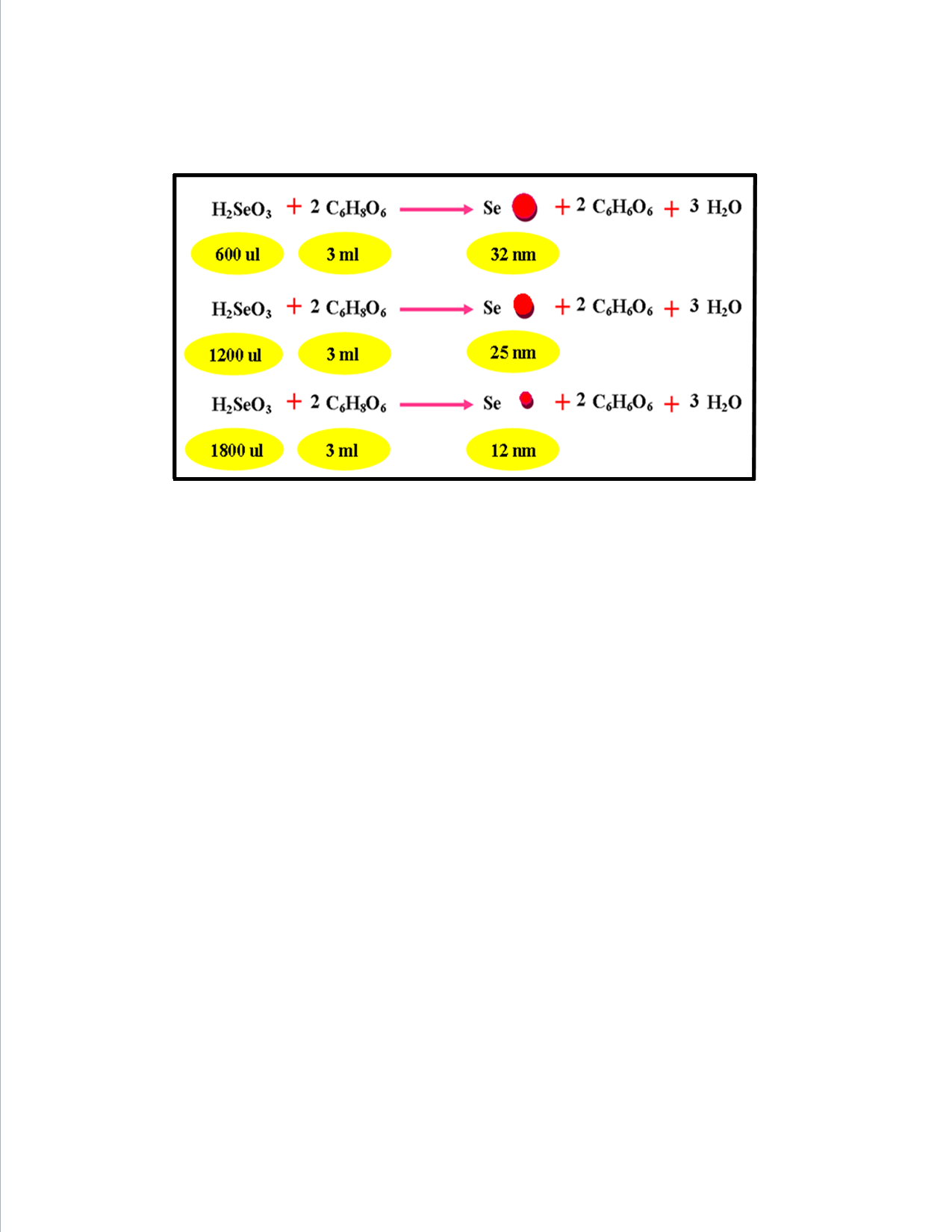


**Figure S1.** Synthesis of Se NPs with different sizes through changing 0.1 M selenous acid volume**.**

**
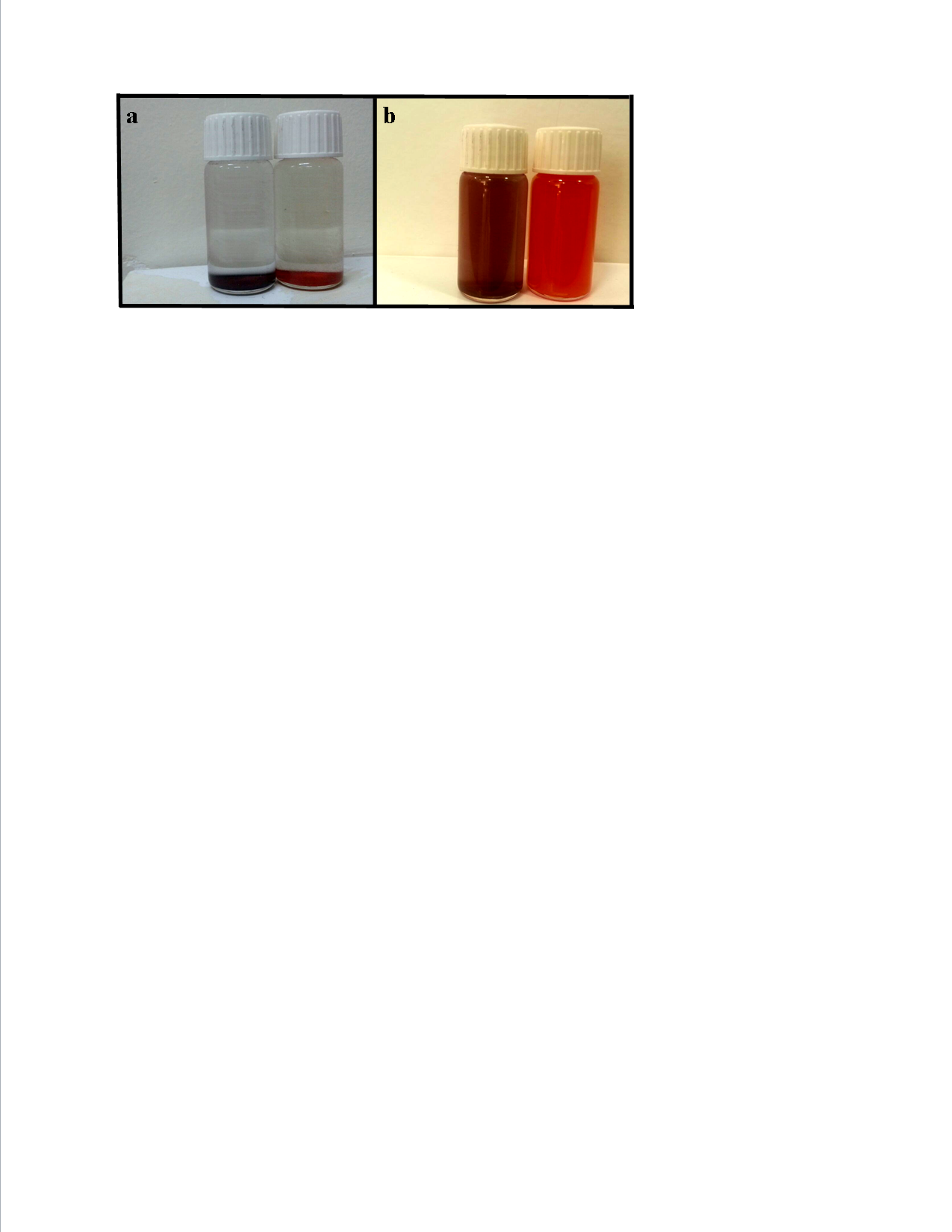
**

**Figure S2.** The higher stability of OX26-PEG-Se NPs as compared to bare NPs. A) Discernible precipitation of bare Se NPs after synthesis (24 h) and B) high stability of OX26-PEG-Se NPs after 7 days.


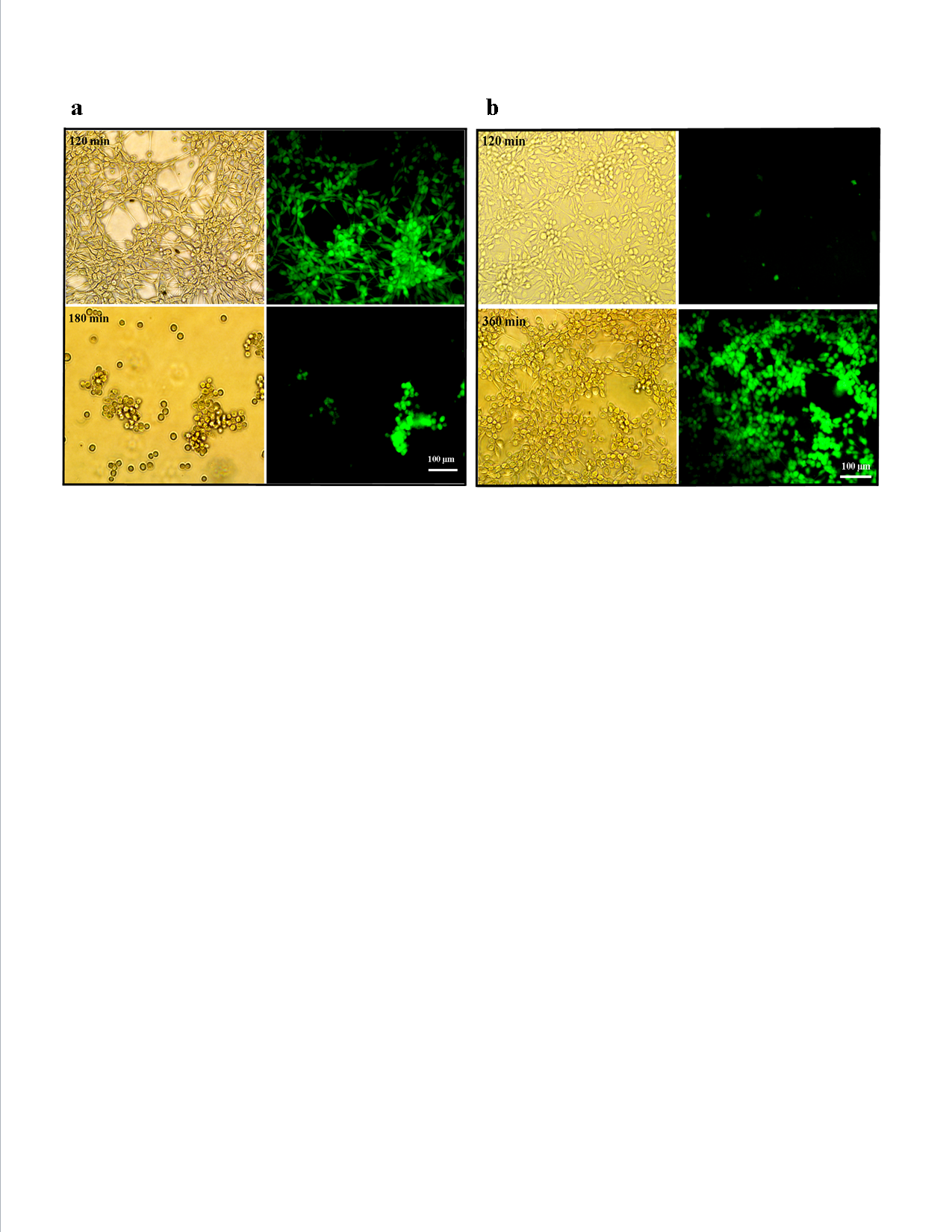


**Figure S3.** Effects of the serum proteins on the NP uptake and cell survival after 30 min OGD for PC12 cells. Bright field (left column in each group) and fluorescence (right column in each group) images of FITC-OX26-PEG-Se NPs (25 μg/mL) A) in SF medium and B) in cMEM. The absence of serum resulted in a significant decrease in the cell number, high NP uptake, loss of the integrity of neurites and cell shrinkage. The presence of serum resulted in decrease of cellular uptake as well as higher cell survival. One may attribute these effects to corona formation and/or the changes in the target cells due to the presence of the serum. The cell-to-cell contacts and cell growth were observable after 360 min in cMEM, suggesting positive effect of corona under oxidative stress condition.


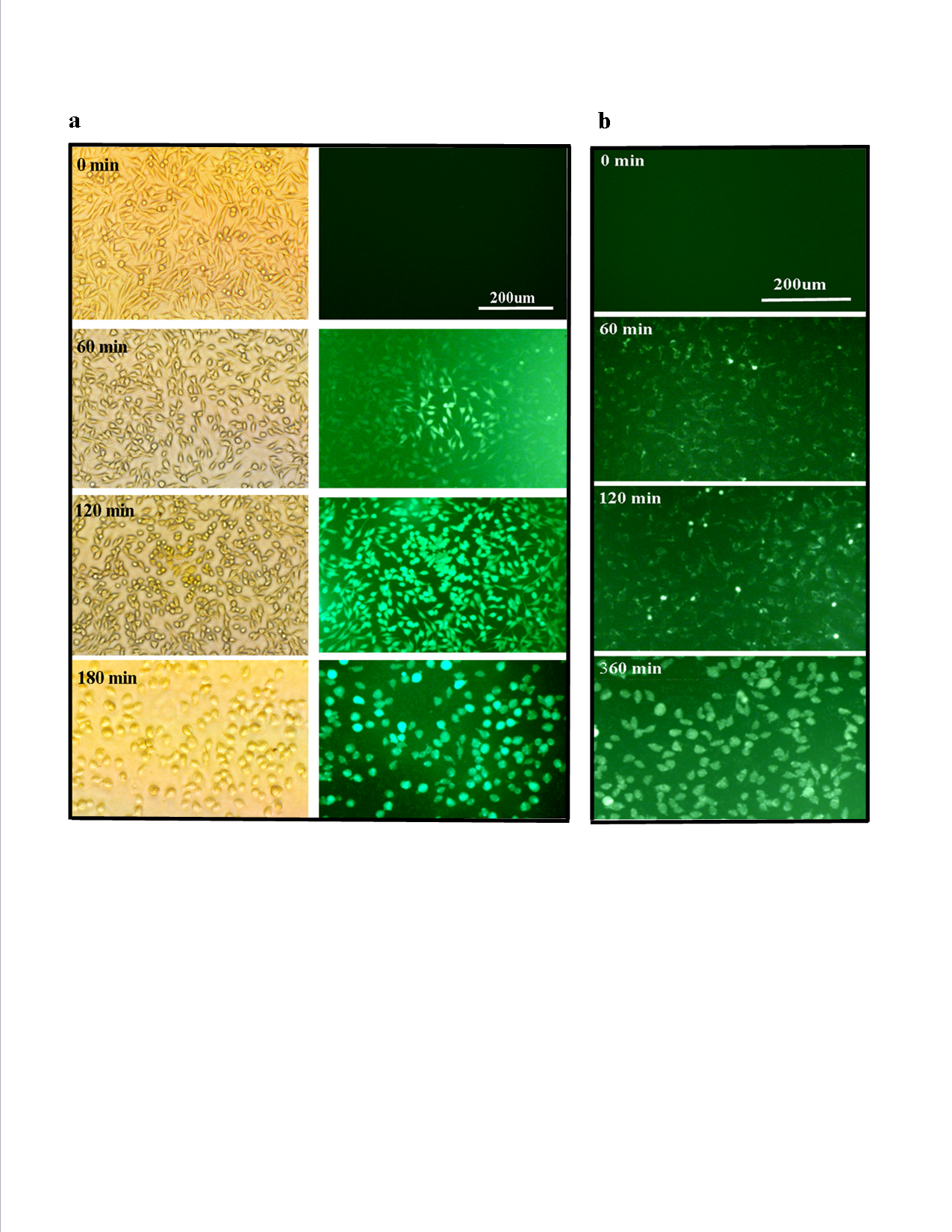


Figure S4. Effects of the presence/absence of protein corona on the NP uptake and cell survival after OGD for MCF7 cells. A) Bright field (left) and fluorescence (right) images of FITC-OX26-PEG-Se NPs in SF medium. The absence of protein corona followed by strong adhesion of particles to the cell membrane resulted in higher uptake and cell damage by 25 μg/mL of the NPs. B) The presence of protein corona not only induces a lower degree of uptake, but also contributes to cell survival exposed to OGD.

**
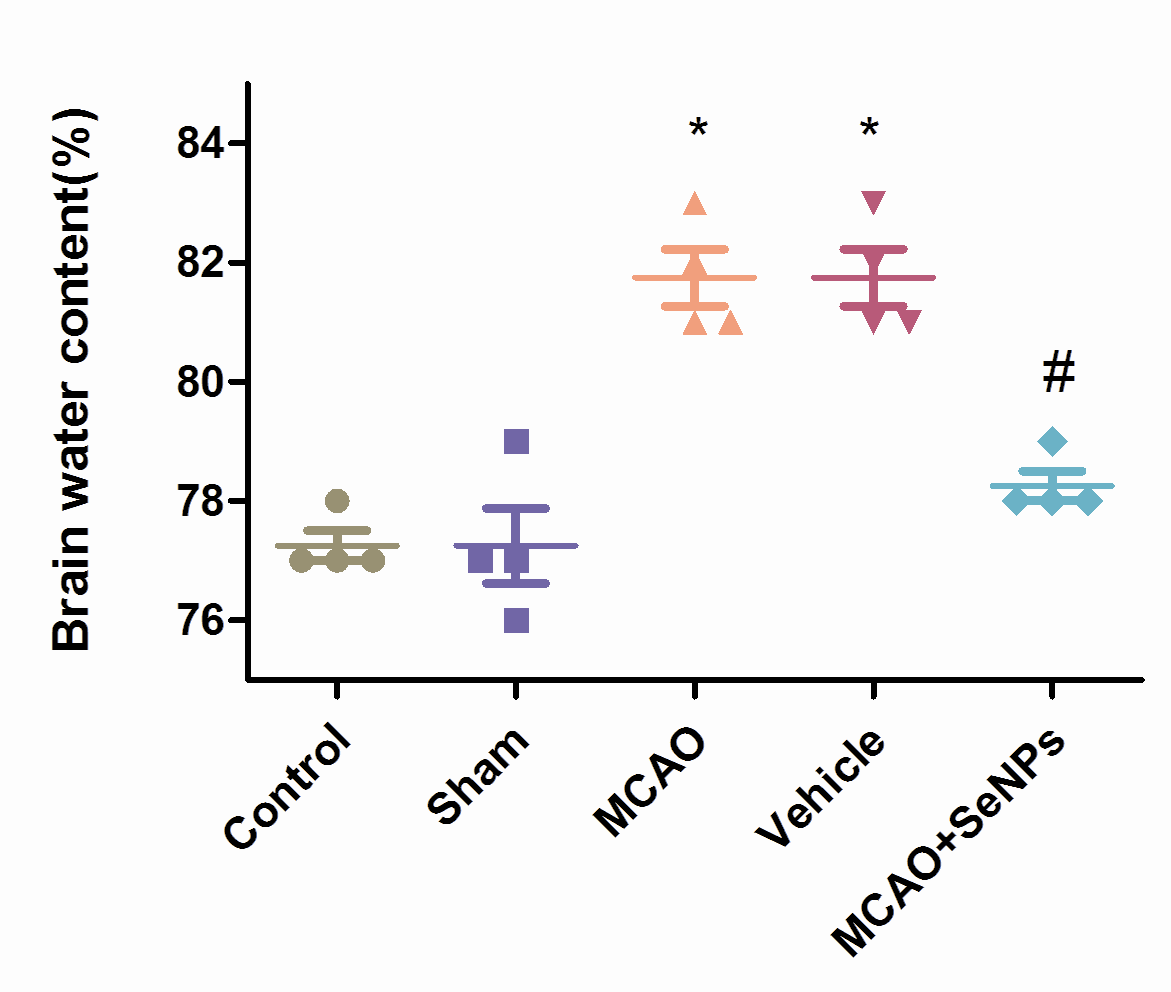
**

**Figure S5.** Intraperitoneal administration of OX26-PEG-Se NPs at dose of 1000 μg/mL significantly reduced brain edema. In contrast, brain water content significantly increased in MCAO and Vehicle cohorts. (*p<0.001 compared to control and sham, # p<0.001 compared to MCAO and vehicle, N = 4 animals per group).

**
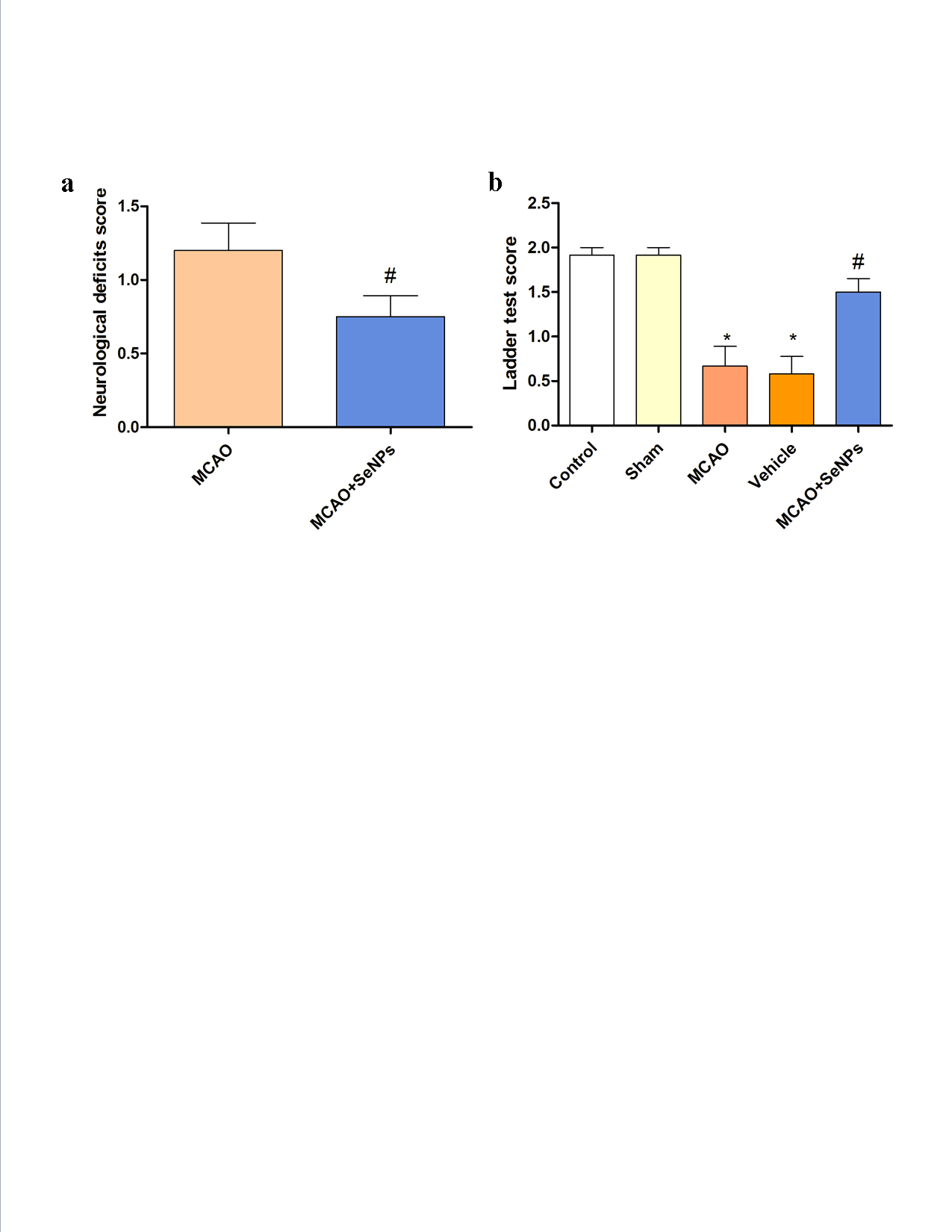
**

**Figure S6.** The OX26-PEG-Se NPs decreased neurological deficits in rats. A) Pretreatment by OX26-PEG-Se NPs significantly reduces neurological deficits (#p<0.01 compared to MCAO). B) Disrupted motor function was observed after stroke (*p<0.001 compared to control and sham group). Pretreatment by OX26-PEG-Se NPs resulted in recovery of motor function following stroke (#p<0.01 compared to MCAO and Vehicle).


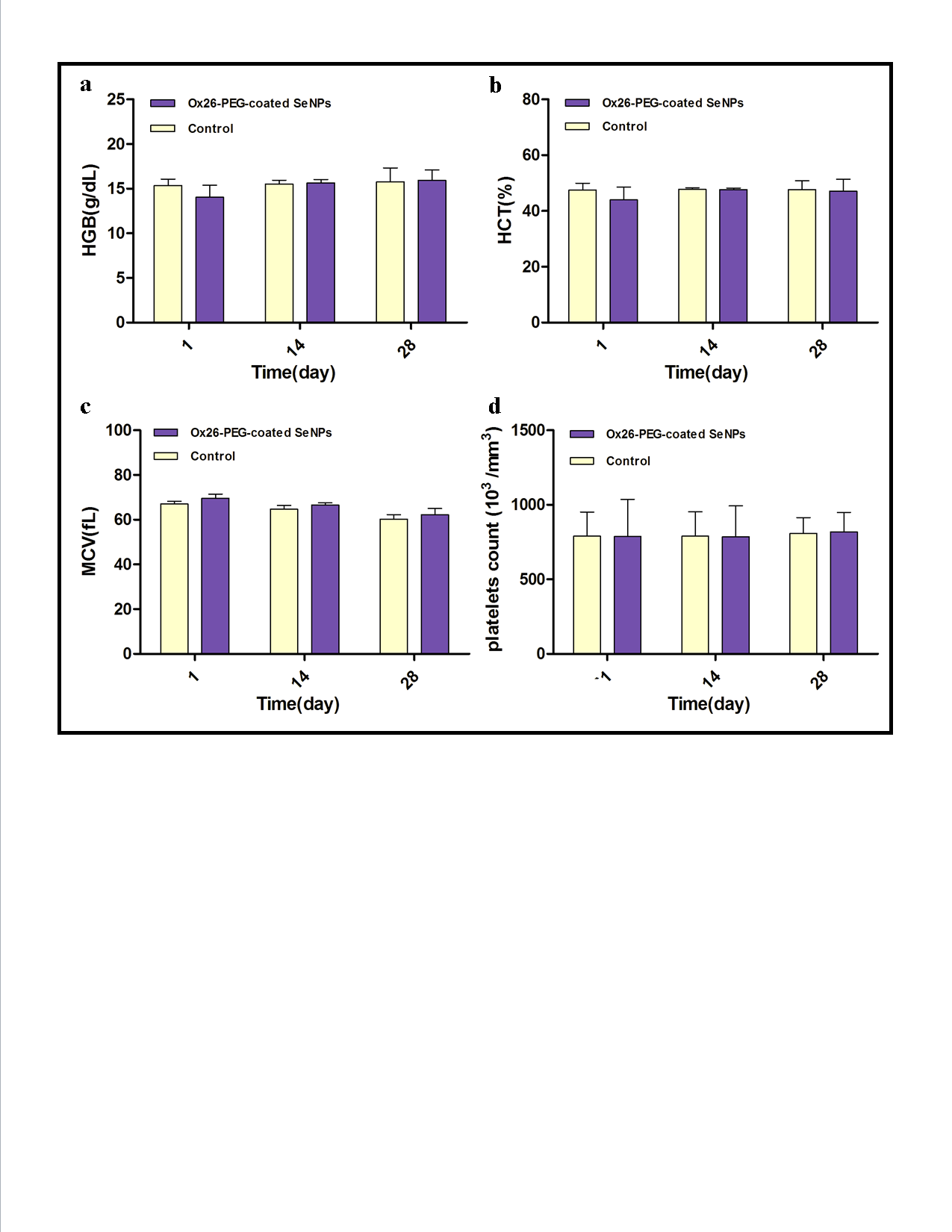


**Figure S7.** Haematological parameters following OX26-PEG-Se NPs intraperitoneal injection (1000 μg/ml at 1, 14 and 42 days). A) The haemoglobin concentration, B) haematocrit volume, C) mean corpuscular volume and D) platelets count did not change compared to control at different times (data expressed as ± s.d, N = 6 animals per group).

**
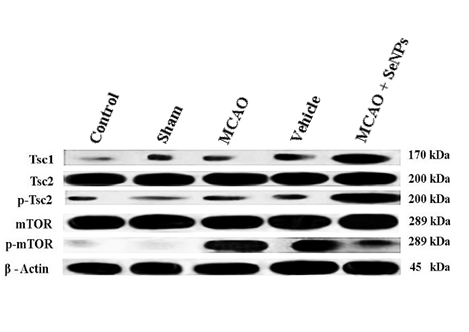
**

**Figure S8. full-length blots for figure 5**

**
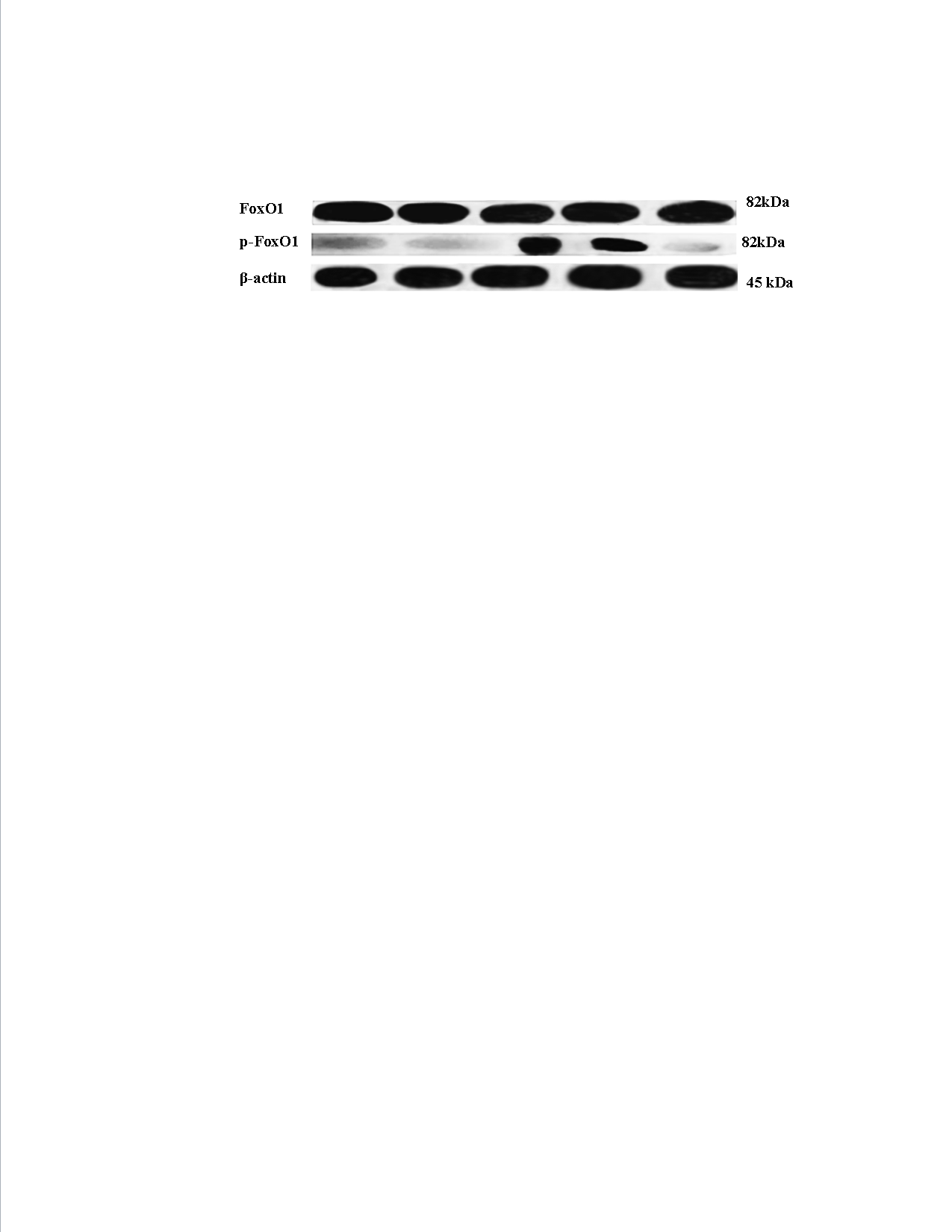
**

**Figure S9. full-length blots for figure 6**

**
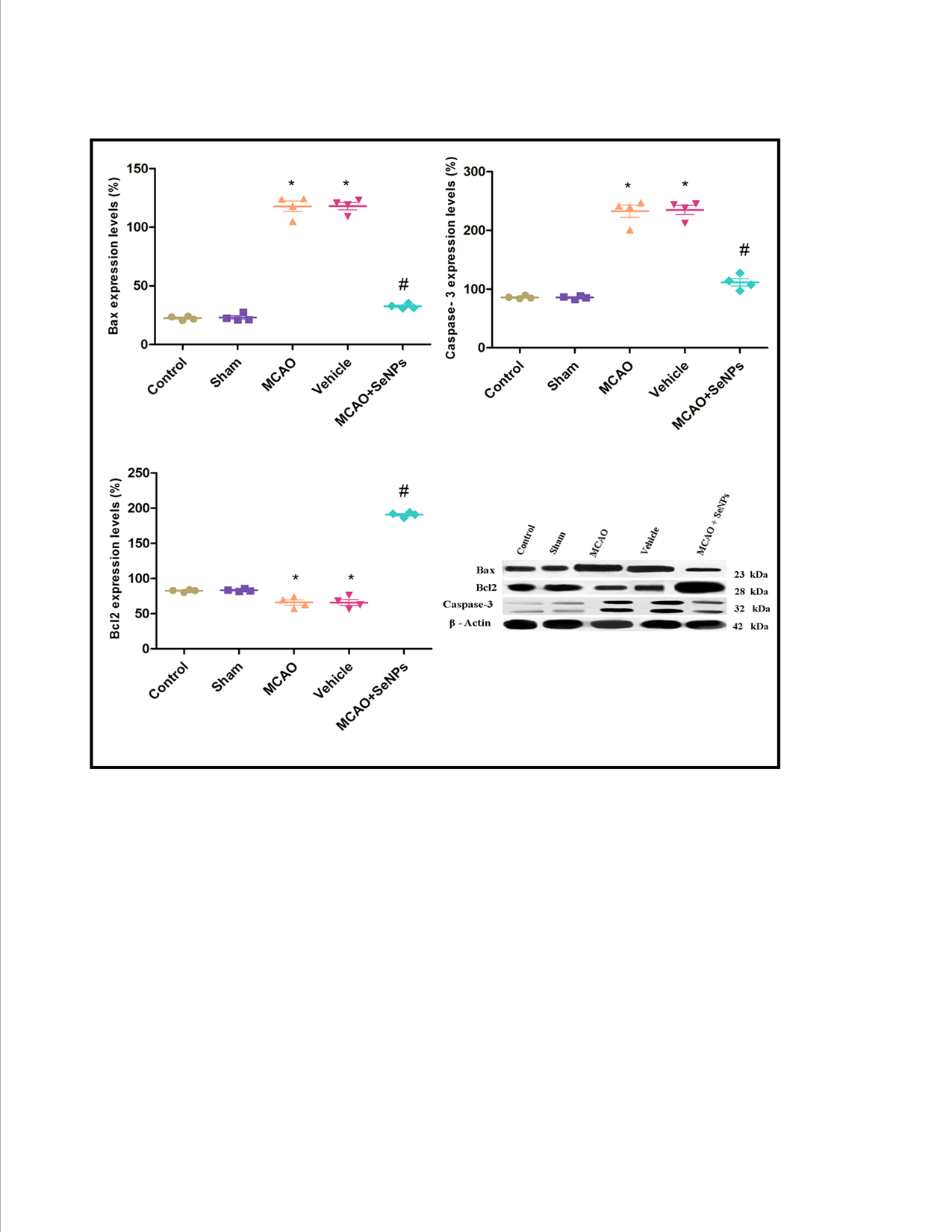
**

**Figure S10. full-length blots for figure 8**

**Table S1.** Quantitation of the OX26 antibody immobilized on the surface of SeNPs

| Added Antibody  Concentration to PEGylated SeNPs (µg/mL) | Corrected  Absorbance* | Concentration of particle in Supernatant (µg/mL)# | Concentration of Adsorbed  Antibody (µg/mL) $ | Average of adsorbed  antibody molecules onto the particle surface** |
| --- | --- | --- | --- | --- |
| 1 | 0.0477±0.0032 | 0.76±0.15 | 0.24±0.15 | 37±15 |
| 5 | 0.1843±0.0078 | 3.11±0.64 | 1.89±0.64 | 261±57 |
| 10 | 0.2646±0.0041 | 8.14±0.28 | 1.86±0.28 | 209± 42 |

*This was based on subtraction of absorbance for the negative control (0 µg/mL

antibody) per Bio-Rad protocol.

# This was based on antibody remaining in the supernatant.

$ This was based on difference in added antibody concentration and antibody remaining in the supernatant

** This was based on dividing of adsorbed antibody (converted to antibody/mL, using antibody MW of 150 K mol. wt) by the concentration of SeNPs (2× 1010 SeNP/mL).

**Table S2.** Physicochemical characteristics of bare Se NPs in 10% human plasma medium.

| **Time (min)** | 10 | 30 | 60 | 120 | 180 |
| --- | --- | --- | --- | --- | --- |
| **Size (nm)** | 40.1 ± 5.1 | 56.4 ± 8 | 24 ± 4.6 | 33.2±3 | 34.2±3 |
| **Polydispersity index** | 0.16±0.02 | 0.15±0.01 | 0.15±0.03 | 0.13±0.03 | 0.14±0.01 |
| **Zeta potential (mV(** | -7.75±1.35 | -5.55±3 | -20.17±1.44 | -20.83± 0.86 | -20.81±1.3 |
| **Mobility (μm cm/(V s))** | -0.61 ± 0.08 | -0.43 ± 0.22 | -1.58 ± 0.2 | -1.63 ± 0.06 | -1.62± 0.03 |

**Table S3.** Physicochemical characteristics of bare Se NPs in cMEM.

| **Time** (min) | 10 | 30 | 60 | 120 | 180 |
| --- | --- | --- | --- | --- | --- |
| **Size** (nm) | 49.30 ±10 | 77.8±6.6 | 39.4±2 | 93.3±12.8 | 92.77±14.1 |
| **Polydispersity index** | 0.14±0.04 | 0.20±0.01 | 0.16±0.05 | 0.15±0.05 | 0.16±0.02 |
| **Zeta potential (mV(** | -13.10±1.30 | -4.36±3.1 | -20.42±3.12 | -7.27±2 | -10.21±1 |
| **Mobility (μm cm/(V s))** | -1.02±0.04 | -0.34±0.03 | -1.60±0.31 | -1.79±0.03 | -1.73±0.02 |

**Table S4.** Physicochemical characteristics of OX26-PEG-Se NPs in 10% human plasma medium.

| **Time (min)** | 10 | 30 | 60 | 120 | 180 |
| --- | --- | --- | --- | --- | --- |
| **Size (nm)** | 38.1±6 | 36.3±6 | 42.4±5 | 33.9± 7.1 | 34.1±4.3 |
| **Polydispersity index** | 0.39±0.01 | 0.46±0.04 | 0.40±0.08 | 0.44±0.06 | 0.41±0.03 |
| **Zeta potential (mV(** | -1.13±2.1 | -1.27±3 | -1.03±1.60 | -1.25±2.76 | -1.37±3.2 |
| **Mobility (μm cm/(V s))** | -0.82±0.02 | -0.93±0.01 | -0.76±0.04 | -0.89±0.03 | -0.91±0.02 |

**Table S5.** Physicochemical characteristics of OX26-PEG-Se NPs in cMEM.

| **Time** (min) | 10 | 30 | 60 | 120 | 180 |
| --- | --- | --- | --- | --- | --- |
| **Size (nm)** | 32. 2±4 | 46±4.8 | 53.4±7 | 25.6±5 | 27.12±3 |
| **Polydispersity index** | 0.17±0.02 | 0.18±0.01 | 0.13±0.01 | 0.11±0.06 | 0.12±0.03 |
| **Zeta potential (mV(** | -4.81±1 | -25.30±1 | -2.20±1.02 | -10.23±1 | -8.23±1.16 |
| **Mobility** **(μm cm/(V s))** | -0.38±0.06 | -1.98±0.04 | -0.17±0.06 | -0.80±0.08 | -0.63±0.03 |

**Table S6.** Primer Sequences For RT-PCR Analysis.

| **Gene** | **Accession No.** | **Sequence (5' to 3')** | **Amplicon** **(bp)** |
| --- | --- | --- | --- |
| Rictor | XM_008775080.2 | (F) TGCTTCCTTGTTTCCGAGTT | 138 |
|  |  | (R) GCTACCACCTCTGGGTTCTG |  |
| Wnt3a | NM_001105715.1 | (F) TGTGAGGTGAAGACCTGCTG | 207 |
|  |  | (R) AAAGTTGGGGGAGTTCTCGT |  |
| β catenin | NM_053357.2 | (F) CTGACCAAACTGCTAAATGACG | 208 |
|  |  | (R) GATGGTGGGAAAGGTTGTGTAG |  |
| Mst1 | NM_001107800.1 | (F) GCTATTTTGTAAGGTTTGGCTGTAAA | 81 |
|  |  | (R) CACGGCAGTGGAGGAAGCT |  |
| Yap1 | NM_001034002.2 | (F) AAGGCTTGACCCTCGTTT | 133 |
|  |  | (R) CTGCTGCTGCTGGTTTGA |  |
| Jak2 | NM_031514.1 | (F) GTGGAGATGTGCCGCTATG | 157 |
|  |  | (R) CCTTGTACTTCACGATGTTGTC |  |
| Stat3 | NM_012747.2 | (F) GAAGAGTGCCTTCGTGGT | 105 |
|  |  | (R) AGCAACCTGACTTTTGTGG |  |
| ERK5 | NM_001191547.1 | (F) CTCCTTCGACGTGACCTTTG | 129 |
|  |  | (R) ATCTTCTTGATGGCCACCTG |  |
| Adamts1 | NM_024400.2 | (F) GCCTACATGGTCACATCATTCCT | 119 |
|  |  | (R) TGGCGGTTGGCATCGTA |  |
| Ulk1 | NM_001108341.1 | (F) GGCTTACAGACTGCCATTGA | 110 |
|  |  | (R) GATACCACGCTGGCCTTATAC |  |
| Gapdh | NM_017008.4 | (F) AGTTCAACGGCACAGTCAAG | 118 |
|  |  | (R) TACTCAGCACCAGCATCACC |  |

**Table S7.** Antibodies for Western assay.

| **Antibody** | **Catalog No.** | **Company** | **Use** | **Molecular Weight** |
| --- | --- | --- | --- | --- |
| Hamartin/TSC1 Antibody | #4906 | Cell Signaling Technology | 1:1000 | 150 to 170 kDa |
| Tuberin/TSC2 Antibody | #3612 | Cell Signaling Technology | 1:1000 | 200 kDa |
| Phospho-Tuberin/TSC2 (Ser1387) Antibody | #5584 | Cell Signaling Technology | 1:1000 | 200 kDa |
| mTOR  Antibody | #2972 | Cell Signaling Technology | 1:1000 | 289 kDa |
| Phospho-mTOR (Ser2481) Antibody | #2974 | Cell Signaling Technology | 1:1000 | 289 kDa |
| FoxO1 (L27) Antibody | #9454 | Cell Signaling Technology | 1:1000 | 78 to 82 kDa |
| Phospho-FoxO1 (Ser256)  Antibody | #9461 | Cell Signaling Technology | 1:1000 | 82 kDa |
| Bax Antibody (N-20) | sc-493 | Santa Cruz Biotechnology | 1 : 2000 | 23 kDa |
| Bcl-2 Antibody (C 21) | sc-783 | Santa Cruz Biotechnology | 1 : 2000 | 28 kDa |
| Anti-Caspase-3 antibody | ab4051 | Abcam | 1 : 500 | 32 kDa |
| β-Actin Antibody | #4967 | Cell Signaling Technology | 1:1000 | 45 kDa |
| Anti-rabbit IgG,  HRP-linked Antibody | #7074 | Cell Signaling Technology | 1:3000 |  |
